# Targeting Mitochondrial Stress Responses: Terbinafine and Miglustat as Novel Lifespan and Healthspan Modulators

**DOI:** 10.1111/acel.70452

**Published:** 2026-03-30

**Authors:** Amélia Lalou, Ioanna Daskalaki, Ilias Gkikas, Sandra Rodríguez‐López, Jean‐David Morel, Giorgia Benegiamo, Adrien Faure, Arwen W. Gao, Joaquim Barmaz, Qi Wang, Terytty Yang Li, Feng Gao, Danaé Broustail, Kristina Schoonjans, Giovanni D'Angelo, Johan Auwerx

**Affiliations:** ^1^ Laboratory of Integrative Systems Physiology, Institute of Bioengineering École Polytechnique Fédérale de Lausanne Lausanne Switzerland; ^2^ Laboratory of Lipid Cell Biology, Institute of Bioengineering École Polytechnique Fédérale de Lausanne Lausanne Switzerland; ^3^ Laboratory of Metabolic Signaling, Interfaculty Institute of Bioengineering École Polytechnique Fédérale de Lausanne Lausanne Switzerland; ^4^ Laboratory Genetic Metabolic Diseases, Amsterdam Gastroenterology, Endocrinology and Metabolism Institute, Amsterdam University Medical Centers University of Amsterdam Amsterdam the Netherlands; ^5^ Shanghai Key Laboratory of Metabolic Remodeling and Health, Laboratory of Longevity and Metabolic Adaptations, Institute of Metabolism and Integrative Biology Fudan University Shanghai China

**Keywords:** aging, *Caenorhabditis elegans*, doxycycline, drug repositioning, longevity, miglustat, mitochondria, terbinafine

## Abstract

Mitochondria are central to cellular homeostasis and play a critical role in aging and age‐related disorders, making them promising therapeutical targets. Here, we identify terbinafine and miglustat as novel mitochondrial stress inducers that extend lifespan and improve healthspan in *Caenorhabditis elegans.* Through a two‐step screening, we found that both compounds activate the mitochondrial stress response (MSR) and exhibit distinct mechanisms of action. Terbinafine and miglustat robustly activated the mitochondrial unfolded protein response (UPRmt) mediator ATFS‐1, upregulated MSR pathways, and modulated mitochondrial function across species, similarly to doxycycline. Interestingly, both compounds also engaged the insulin/IGF‐1 signaling (IIS) pathway in *C. elegans* , revealing an integrated stress response involving coordinated action of ATFS‐1 and the FOXO transcription factor DAF‐16, distinct from canonical IIS activation. Experiments in human HEK293T cells confirmed the translational potential, with both compounds inducing mitochondrial stress and modulating mitochondrial function in mammalian systems. This study highlights the potential of harnessing the MSR to promote longevity and mitigate age‐related functional decline. The identification of terbinafine and miglustat as mitochondrial stressors paves the way for novel anti‐aging therapies.

## Graphical abstract

Terbinafine and miglustat are mitohormetic compounds that extend lifespan and healthspan. In *C. elegans* , these FDA‐approved drugs engage both ATFS‐1 and DAF‐16, highlighting a stress‐dependent coordination between mitochondrial and IIS pathways distinct from canonical IIS activation, relevant for healthy aging.

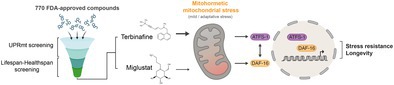

## 1 Introduction

Aging is characterized by a gradual functional decline and increased susceptibility to a variety of non‐communicable diseases, including cancer, cardiovascular, and neurodegenerative disorders. Current therapies merely address the symptoms, without tackling the underlying pathophysiology of these diseases. With the aging population projected to reach 2.1 billion by 2050 (World Health Organization), there is an urgent need for interventions promoting healthy aging.

Age‐related diseases share numerous biological impairments, including genomic instability, telomere attrition, epigenetic alterations, loss of proteostasis, deregulated nutrient‐sensing, cellular senescence, stem cell exhaustion, altered intercellular communication, and mitochondrial dysfunction (López‐Otín et al. 2023). Among these, mitochondrial dysfunction has emerged as a key driver of aging and disease progression (Gorman et al. 2016). Mitochondria are essential organelles participating in numerous cellular functions, including energy harvesting, biogenesis, regulation of homeostasis and apoptosis (Martínez‐Reyes and Chandel 2020). Changes in mitochondrial integrity not only impact cellular metabolism but also critically influence whole‐body metabolism, health, and lifespan. Consequently, mitochondrial‐targeted therapies have gained significant attention for treating metabolic and age‐related conditions.

One promising approach is the pharmacological induction of the mitochondrial stress response (MSR), an adaptive pathway that restores proteostasis and promotes resilience to stress. While severe mitochondrial dysfunction is detrimental, mild mitochondrial stress can extend lifespan and delay age‐related decline, a phenomenon known as mitohormesis (Mottis et al. 2022). MSR‐inducing compounds have shown potential in mitigating age‐related decline and improving outcomes in various conditions (Mottis et al. 2022; Romani et al. 2021; Sorrentino et al. 2017).

A key component of the MSR is the mitochondrial unfolded protein response (UPRmt) (Durieux et al. 2011), which coordinates cellular responses to mitochondrial stress and maintains mitochondrial proteostasis. In *C. elegans* , the UPRmt is initiated by misfolded proteins, leading to the activation of the transcription factor associated with stress 1 (ATFS‐1), which induces chaperones, proteases, and metabolic regulators to re‐establish mitochondrial homeostasis (Jovaisaite et al. 2014; Nargund et al. 2012). Similar mechanisms are observed in mammals, where ATF4 and ATF5 mediate mitochondrial stress responses (Mottis et al. 2019). Notably, mild mitochondrial perturbations, including mitochondrial ribosomal protein knockdown or antibiotic treatment, like doxycycline, can activate the UPRmt and extend lifespan in *C. elegans* and other species (Houtkooper et al. 2013). Doxycycline‐induced MSR has also been shown to ameliorate proteostasis in Alzheimer's (Sorrentino et al. 2017) and muscle amyloidosis (Romani et al. 2021) models, and improve survival in lethal influenza infection (Mottis et al. 2022).

Beyond mitohormesis, the insulin/IGF‐1 signaling (IIS) pathway is another key longevity regulator (Zečić and Braeckman 2020). In *C. elegans* , IIS is triggered by insulin‐like peptides binding to DAF‐2, the homolog of the insulin/IGF‐1 receptor. The activation of DAF‐2 initiates a phosphorylation cascade leading to the inactivation of DAF‐16, a FOXO transcription factor essential for stress resistance and lifespan extension (Oh et al. 2005). Conversely, reduced IIS leads to the nuclear accumulation of DAF‐16 which promotes longevity (Hsu et al. 2003; Murphy and Hu 2013). Interestingly, DAF‐16 is also required for lifespan extension in long‐lived mitochondrial mutants (Senchuk et al. 2018), suggesting a crosstalk between MSR and IIS that remains incompletely understood.

Despite progress in aging research, few pharmacological agents robustly activate the MSR without adverse effects. While antibiotics like doxycycline robustly induce the UPRmt, their antibacterial activity disrupts the microbiome and contributes to antibiotic resistance, limiting their therapeutic potential. Thus, identifying mitochondrial stress inducers without antibacterial properties is crucial.

Here, we screened 770 FDA‐approved drugs to identify novel MSR activators. Using *C. elegans* , we identified terbinafine and miglustat as mitochondrial stress modulators that extend lifespan and healthspan without antibacterial activity. Mechanistically, both compounds activate the UPRmt and engage DAF‐16‐dependent IIS signaling, distinct from its canonical activation, revealing a coordinated stress adaptation program. Importantly, terbinafine and miglustat also induce mitochondrial stress responses in human cells, supporting their translational relevance and highlighting new opportunities to target mitochondrial dysfunction in aging.

## 2 Results

### 2.1 Comprehensive Screening Identifies Terbinafine and Miglustat as Novel Mitochondrial Stress Modulators That Enhance Lifespan and Healthspan

**FIGURE 1:**
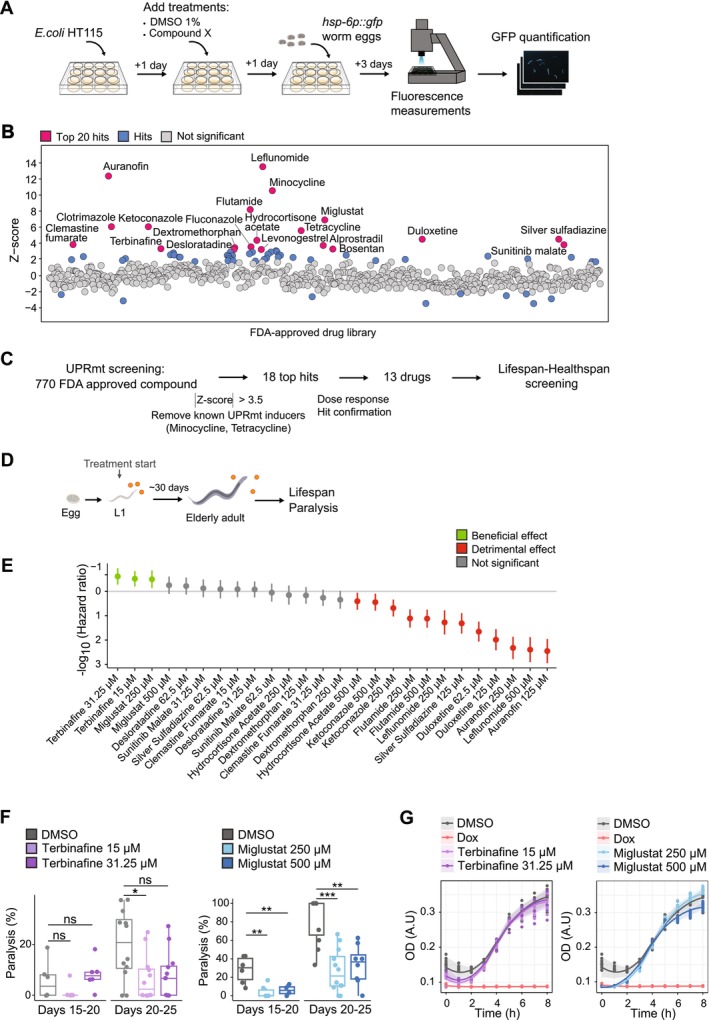
Screening identifies terbinafine and miglustat as mitochondrial stress inducers that extend lifespan and improve healthspan. (A) Flowchart of the UPRmt screening using *hsp‐6p::gfp* reporter strain in 12‐well plates. (B) *Z*‐score distribution of all tested compounds. The top 20 hits are highlighted in pink (abs(*Z*‐scores) > 3.5). Compounds with significant *Z*‐scores are indicated in blue, while non‐significance is shown in gray. (C) Screening pipeline illustrating the screening process, starting with a library of 770 FDA‐approved compounds, narrowing down to 18 novel hits, out of which 13 were confirmed and selected for further characterization. (D) Lifespan and healthspan screening strategy. Compounds were administered from L1 stage, lifespan and paralysis rates were continuously monitored. (E) Graphical analysis of longevity. The impact of each drug is quantified using the negative logarithm (base 10) of the hazard ratio (HR). The effects of the compounds are color‐coded for clarity, with green indicating lifespan extension, gray indicating no effect, and red indicating lifespan reduction relative to DMSO‐treated worms. (F) Box‐plot depicting the percentage of paralysis in terbinafine‐ and miglustat‐treated worms between days [15–20] and [20–25]. Statistical significance, determined after FDR correction, is indicated (*p < 0.05, **p < 0.01, ***p < 0.001). (G) Growth curves of OP50 *E. coli* in the presence of terbinafine (15 μM, 31.5 μM), miglustat (250 μM, 500 μM) or doxycycline (Dox) (50 μM).

To identify novel MSR‐activating compounds, we screened 770 FDA‐approved drugs, using the *C. elegans* reporter strain *hsp‐6p::gfp*, which mainly reflects UPRmt activation via ATFS‐1 (Figure 1A). Compounds were tested at 100 μM or 50 μM if toxicity was observed. We identified 69 hits (abs(Z) > 1.96; Figure 1B) and prioritized the top 20 (*Z* > 3.5; Figure 1C), including known UPRmt activators (tetracyclines), validating the screen. Since tetracyclines' mechanism of action on healthspan is well characterized, they were excluded from further analysis (Houtkooper et al. 2013; Mottis et al. 2022). Interestingly, the remaining 18 compounds spanned diverse therapeutic classes, including anti‐inflammatory, antifungal, cardiovascular, neuropsychiatric, and antiallergic agents (Table S1), highlighting the broad range of pharmacological interventions capable of modulating mitochondrial function.

Mild mitochondrial perturbation can promote longevity through hormetic mechanisms (Lima et al. 2022). However, excessive stress can be detrimental, emphasizing the importance of identifying compounds that operate within a beneficial hormetic window (Lima et al. 2022). To identify concentrations sufficient to induce the MSR while limiting potential toxicity, we performed a dosage titration using the *hsp‐6p::gfp* reporter strain and validated 13 compounds (Figure S1). To evaluate their effects on longevity, we tested two concentrations per compound: the lowest dose capable of inducing MSR activation during titration and a lower concentration to mitigate potential toxicity from chronic exposure.

Lifespan analysis revealed striking differences among the compounds (Figures 1E and S2, Table S2). While seven compounds reduced lifespan, four had no significant effect, and two significantly extended lifespan.

Terbinafine, an antifungal agent that inhibits squalene epoxidase, increased mean lifespan by 9.2% (31.25 μM; hazard ratio = −0.62, *p* < 0.001) and 7.3% (15 μM; hazard ratio = −0.52, *p* < 0.01). Miglustat, a glucosylceramide synthase inhibitor, extended mean lifespan by 5.5% at 250 μM (hazard ratio = −0.50, *p* < 0.01); its effect at 500 μM was weaker (2.6%, hazard ratio = −0.25, *p* = 0.16).

Aging is often accompanied by sarcopenia, the progressive loss of muscle mass and function, which contributes to frailty and reduced quality of life (Beaudart et al. 2023). To assess healthspan, we measured age‐related paralysis (Days 20–25) in *C. elegans* , a marker of muscle decline (Statzer et al. 2022). Both compounds reduced paralysis, with maximal reductions of 60% for terbinafine and ~40% for miglustat (Figure 1F).

A key limitation of many UPRmt inducers is their antibacterial activity, which limits their therapeutic potential. In contrast, terbinafine and miglustat did not show detectable antibacterial activity against *E. coli* OP50 under our experimental conditions (Figure 1G). Moreover, chronic treatment did not affect body size or egg‐laying capacity (Figure S3), which is different from the effects of doxycycline. These findings suggest that the lifespan and healthspan improvements observed are unlikely to be driven by developmental or reproductive defects. This sets terbinafine and miglustat apart from many previously identified mitohormetic agents and highlights their potential for translational applications in aging and age‐related diseases.

### 2.2 Terbinafine and Miglustat Activate a Mitochondrial Stress Response Similar to Doxycycline

**FIGURE 2:**
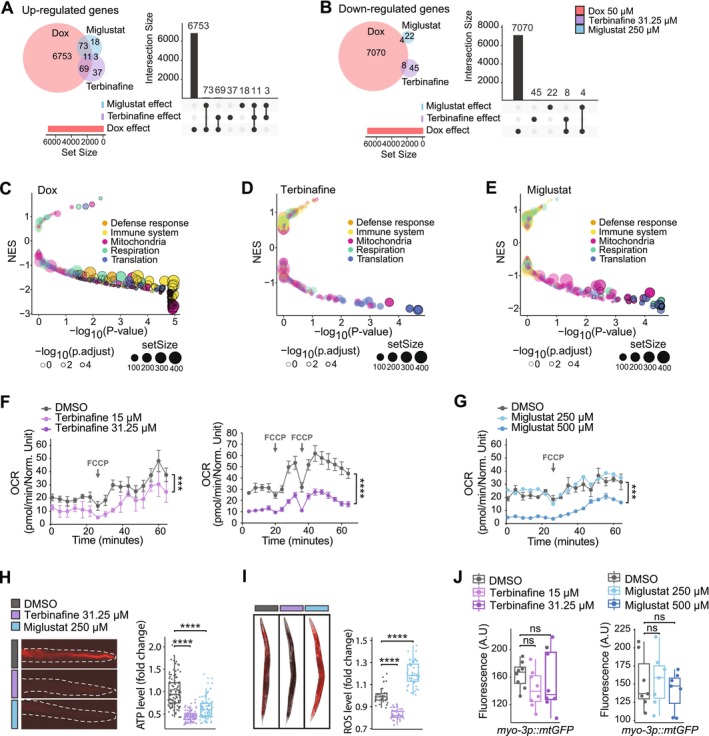
Terbinafine and miglustat activate mitochondrial stress response and modulate mitochondrial function. (A, B) Transcriptional overlaps between doxycycline (Dox) (50 μM), miglustat (250 μM) and terbinafine (31.5 μM). (A) Up‐regulated genes; (B) Down‐regulated genes. (C–E) Volcano plots representation of GSEA Normalized Enrichment Scores (NES) for doxycycline (Dox) (50 μM), terbinafine (31.5 μM) and miglustat (250 μM) treatments, highlighting pathways related to mitochondrial function, translation and immune response. (F, G) Oxygen consumption rate (OCR) measurements in Day 1 adult worms treated with terbinafine, miglustat or vehicle. (H) ATP content measurements in Day 1 adult worms treated with terbinafine, miglustat or vehicle. (I) ROS measurements in Day 1 adult worms treated with terbinafine, miglustat or vehicle. (J) GFP quantification of mitochondrial content in *myo‐3p::mtGFP* reporter worms, at Day 1 of adulthood, following terbinafine or miglustat supplementation.

To gain insight into the mechanisms by which terbinafine and miglustat promote longevity, we performed transcriptome analysis in Day 1 adult worms. Doxycycline induced extensive transcriptional changes (6908 genes up‐regulated, 7082 genes down‐regulated), while terbinafine and miglustat elicited more targeted responses (120 and 105 genes up‐regulated, 53 and 26 genes down‐regulated, respectively; Figure 2A–B). Despite these differences in magnitude, up‐regulated genes showed substantial overlap with Doxycycline (67% for terbinafine, 80% for miglustat) (Figure 2A), suggesting a shared activation of specific pathways. In contrast, down‐regulated genes showed less overlap (17.8% and 18.2%, respectively) (Figure 2B). Interestingly, 11 genes were consistently induced across all treatments and were enriched for fatty‐acid metabolism and proteostasis, including the mitochondrial protease *lonp‐2* (Table S3).

To better characterize the specificity of terbinafine and miglustat, we also explored the genes exclusively up‐regulated by each compound (Tables S4–S6). Terbinafine‐specific genes revealed pathways related to oxidant detoxification, monooxygenase activity and regulation of proteostasis, reflecting its role in promoting protein quality control and protecting against oxidative damage (Table S4).

Interestingly, terbinafine and miglustat share three up‐regulated genes not induced by doxycycline, associated with fatty acid alpha‐oxidation and peroxisome function (Table S6). The shared up‐regulation of these pathways suggests that both compounds increased lipid metabolism and peroxisomal stress responses, which may contribute to their longevity effects. Overall, this analysis highlights terbinafine and miglustat as novel UPRmt inducers that promote cellular and metabolic adaptation.

To further explore the functional implications of these transcriptional changes, we performed gene set enrichment analysis (GSEA). Similar to doxycycline, both terbinafine and miglustat down‐regulated genes linked to mitochondrial function and translation (Figure 2C–E). This transcriptional response was supported by functional mitochondrial readouts. Both compounds reduced basal and maximal respiration as well as ATP levels (Figure 2F–H). Terbinafine decreased mitochondrial reactive oxygen species (mtROS) levels, whereas miglustat modestly increased mtROS (Figure 2I), indicating compound‐specific stress signatures. Neither transcriptional analysis nor in vivo assessment using the *mtRosella* reporter revealed increased mitophagy (Figure S4A–D), arguing against mitochondrial turnover as a driver of the observed effects. Importantly, mitochondrial content was unchanged, as assessed using the *myo‐3p::mtGFP* reporter strain (Figure 2J). Together, these data indicate that terbinafine and miglustat induce mitochondrial stress characterized by reduced mitochondrial function without altering mitochondrial abundance.

### 2.3 Terbinafine and Miglustat Modulate ATFS‐1 Expression and Its Transcriptional Response

**FIGURE 3:**
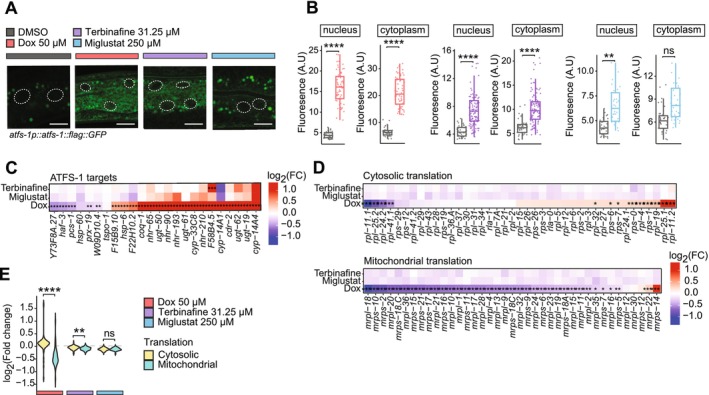
Activation of ATFS‐1 following treatment with doxycycline, terbinafine, and miglustat. (A) Microscopic images of proximal intestinal cells in *atfs‐1p::atfs‐1::flag::gfp* worms treated with control, doxycycline (50 μM), terbinafine (31.5 μM), or miglustat (250 μM). Nuclei are outlined with white dashed circles, and auto‐fluorescent lysosomes appear as punctate structures. Scale bars = 10 μm. (B) Fluorescence quantification of ATFS‐1 expression in the nucleus and cytoplasm (*n* > 10 biological replicates). Statistical significance was assessed using one‐way ANOVA with Bonferroni correction (*p < 0.05, **p < 0.01, ***p < 0.001, ****p < 0.0001). Scale bar = 10 μm. (C) Heatmap of ATFS‐1 targets gene expression across treatments. Expression values are represented in log_2_ fold changes (log_2_FC). Statistical significance after FDR correction is indicated (*p < 0.1, **p < 0.05, ***p < 0.01). (D) Heatmaps of the marker genes for cytosolic and mitochondrial translation following treatments. Expression values are in log_2_ fold changes (log_2_FC). Significance after FDR correction is indicated (*p < 0.1, **p < 0.05, ***p < 0.01). (E). Violin plot representing log_2_ fold changes of genes (log_2_FC) related to cytosolic and mitochondrial function, with statistical significance determined by one‐way ANOVA and Bonferroni correction (*p < 0.1, **p < 0.05, ***p < 0.01, ****p < 0.001).

The UPRmt is a central protective response to mitochondrial stress, orchestrated by the transcription factor ATFS‐1 (Nargund et al. 2015), and has been implicated in the mitohormetic effects of doxycycline (Houtkooper et al. 2013). To determine the role of ATFS‐1 in the effects of terbinafine and miglustat, we used a *C. elegans* strain expressing a GFP‐flagged version of ATFS‐1 under the control of the endogenous *atfs‐1* promoter. Consistent with previous studies (Kaufman and Crowder 2015; Nargund et al. 2012), doxycycline robustly increased ATFS‐1 levels (~3.7‐fold). Terbinafine also elevated total ATFS‐1 expression, although to a lesser extent (~1.7‐fold increase). Similarly, miglustat led to a modest increase in ATFS‐1 levels (Figure 3A,B).

To further validate the activation of the ATFS‐1 and the UPRmt pathway, we examined published RNA‐seq and ChIP‐seq datasets (Nargund et al. 2015) that profile ATFS‐1 activity under mitochondrial stress conditions. Analysis revealed a modest increase in the expression of ATFS‐1 target genes following treatment with all three compounds, with doxycycline eliciting the strongest response, followed by terbinafine and miglustat (Figure 3C). Given the connection between mitochondrial stress and translation regulation, we also investigated the expression of mitochondrial and cytosolic translation‐related genes. Doxycycline strongly suppressed the expression of mitochondrial translation genes compared to cytosolic translation genes. In contrast, terbinafine exhibited a more modest suppression of mitochondrial translation genes, while miglustat had no effect on the balance between mitochondrial and cytosolic translation gene expression (Figure 3D,E). Collectively, these findings confirm that both compounds trigger the canonical UPRmt arm of the broader MSR, although to a lesser extent than doxycycline. This suggests that terbinafine and miglustat may also extend lifespan and improve healthspan through additional regulatory mechanisms beyond the canonical UPRmt activation.

### 2.4 Multiple Stress Response Pathways Contribute to the Effects of Terbinafine and Miglustat

**FIGURE 4:**
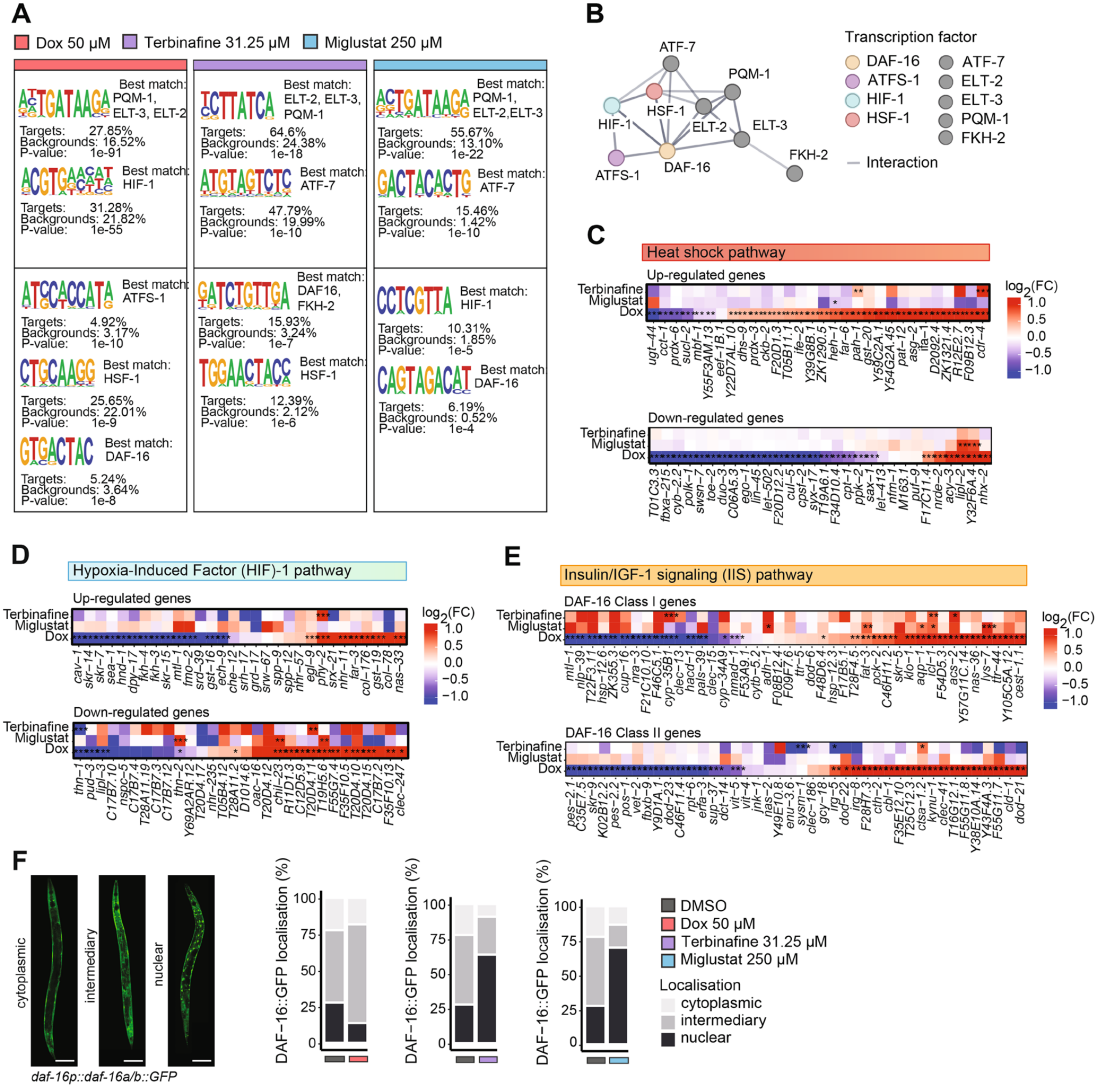
Transcription factor motif analysis reveals that terbinafine and miglustat activate the insulin/IGF‐1 signaling (IIS) pathway. (A) HOMER‐based motif enrichment analysis in genes up‐regulated by doxycycline (50 μM), terbinafine (31.5 μM), and miglustat (250 μM). The column ‘Best match’ signifies the transcription factor that most closely corresponds to the enriched motif. Adjusted *P*‐values are provided. (B) STRING (http://string‐db.org) network visualization of DAF‐16, ATFS‐1, HIF‐1, HSF‐1, ATF‐7, ELT‐2, ELT‐3, and PQM‐1. Colored nodes represent key transcription factors associated with various cellular stress responses, with additional interacting proteins displayed in gray. (C, D) Heatmap of the expression of heat shock specific (C) and Hypoxia‐Inducible Factor (HIF)‐1 (D) genes signature. Expression values are represented in log_2_ fold changes (log_2_FC). Statistical significance after FDR correction is indicated (*p < 0.1, **p < 0.05, ***p < 0.01). (E) Heatmap illustrating the expression patterns of Class I and Class II target genes of DAF‐16. Log_2_ fold changes (log_2_FC) in gene expression are illustrated, with stars indicating levels of significance after FDR correction (*p < 0.1, **p < 0.05, ***p < 0.01). (F) Representative confocal images of DAF‐16 nuclear translocation in *daf‐16p::daf‐16a/b::GFP* worms. The histogram represents the proportion of the number of worms with cytosolic, intermediate, or nuclear localization of DAF‐16 (*n* ≥ 8). Scale bars = 75 μm.

To further dissect the mechanisms underlying terbinafine and miglustat effects, we performed transcription factor motif enrichment analysis on up‐regulated genes using HOMER (Figure 4A, S5A). Genes up‐regulated by doxycycline were enriched with motifs for GATA transcription factors (PQM‐1, ELT‐2, ELT‐3), hypoxia‐inducible factor HIF‐1, ATFS‐1, HSF‐1, and DAF‐16 motifs. Terbinafine‐induced genes showed similar enrichment for GATA transcription factors, ATF‐7, DAF‐16, and HSF‐1 motifs, while those influenced by miglustat exhibited enrichment for GATA transcription factors, ATF‐7, HIF‐1, and DAF‐16 motifs.

Mitochondrial integrity (*atfs‐1*), heat shock (*hsf‐1*), hypoxia (*hif‐1*), and insulin/IGF‐1 signaling (*daf‐16*) represent major cellular stress response pathways that activate protective genes to maintain both cellular and mitochondrial homeostasis (Zečić and Braeckman 2020). Protein–protein interaction analysis revealed a central regulatory network centered around DAF‐16, suggesting a coordinated regulation of multiple stress response pathways that maintain both mitochondrial function and overall cellular homeostasis through the activation of protective genes (Figures 4B and S5B–D).

Given the complex interplay between these stress responses, to discriminate their individual contributions to the compounds' effects, we generated gene signatures from published datasets for the heat shock, hypoxia and the IIS responses. The heat shock response pathway, regulated by HSF‐1, responds to protein misfolding and mitochondrial dysfunction, leading to increased expression of heat shock proteins (HSPs) (Williams et al. 2020). Using published transcriptomic data of heat stress effects on *C. elegans* gene expression (Brunquell et al. 2016), we constructed a heat shock stress response signature comprising both the most significantly up‐regulated and down‐regulated genes. Analysis of this signature revealed that only doxycycline activated this pathway, indicated by an increased expression of heat shock‐induced genes and a decreased expression of heat shock‐suppressed genes (Figure 4C). In contrast, terbinafine and miglustat showed no impact on heat shock response genes. Consistent with this, neither terbinafine nor miglustat induced the heat‐shock reporter *hsp‐16.2p::GFP* in vivo under control conditions (Figure S6A).

Next, we investigated the hypoxia stress response pathway, given the enrichment of HIF‐1 motifs in doxycycline and miglustat‐treated conditions. The hypoxia stress response, orchestrated by HIF‐1 across various organisms, including *C. elegans* , is a crucial adaptive mechanism ensuring cellular survival under low oxygen conditions. Using a published transcriptomic dataset of *C. elegans* subjected to hypoxia (Vora et al. 2022), we built a HIF‐1 gene signature and, similarly, despite the enrichment of HIF‐1 motifs, we found no consistent activation of this pathway by any of the compounds (Figure 4D). This was further supported in vivo using the hypoxia reporter *nhr‐57p::GFP*, which remained unchanged under all treatments under control conditions (Figure S6B).

Finally, given the enrichment of DAF‐16 motifs in all three compound treatments, we examined the insulin/IGF‐1 signaling (IIS) pathway, a major determinant of longevity (Zečić and Braeckman 2020). Reduced IIS has been associated with increased longevity in diverse species, including *C. elegans* (Kenyon et al. 1993), *D. melanogaster* (Clancy et al. 2001), *M. musculus* (Blüher et al. 2003), and in humans, where individuals with Laron syndrome show enhanced protective pathways despite growth hormone (GH)‐insulin‐like growth factor‐1 (IGF1) deficiency (Werner and Laron 2023). Interestingly, DAF‐16 has also been implicated as a downstream mediator of lifespan extension in the long‐lived mitochondrial mutants *clk‐1*, *isp‐1*, and *nuo‐6* (Senchuk et al. 2018), suggesting its role in various conditions of mitochondrial stress. To further explore the DAF‐16 mediated longevity effect, we used a published dataset that categorizes its target genes into two classes based on their expression patterns. Class I genes are associated with stress resistance and longevity, while Class II genes, which are involved in growth and development, are often down‐regulated to promote longevity (Murphy et al. 2003; Tepper et al. 2013). We found that terbinafine and miglustat treatment consistently up‐regulated Class I and down‐regulated Class II genes, indicating a pro‐longevity transcriptional profile. Doxycycline, however, showed variable effects on these gene classes, suggesting that it does not engage the IIS pathway in the same way (Figure 4E). Direct in vivo visualization using the *daf‐16p::daf‐16a/b::GFP* reporter strain confirmed this finding, as terbinafine and miglustat induced nuclear translocation of DAF‐16 in 64% and 70% of treated animals, respectively, compared to 28% in controls (Figure 4F).

Finally, to complement transcriptome‐based inference, we assessed additional stress reporters. Terbinafine moderately increased *sod‐3p::GFP* fluorescence, suggesting a modest activation of oxidative stress defenses, whereas neither compound activated the ER stress reporter *hsp‐4p::GFP* (Figure S6C,D).

Together, these data indicate that terbinafine and miglustat engage IIS/DAF‐16 in addition to mitochondrial stress signaling, whereas doxycycline predominantly activates the canonical UPRmt.

### 2.5 Terbinafine and Miglustat Highlight the Complex Interplay Between ATFS‐1 and DAF‐16 in the Mitochondrial Stress Responses

To test whether ATFS‐1 and DAF‐16 are required for the pro‐longevity effects of terbinafine and miglustat, we performed lifespan assays in *atfs‐1*(gk3094), *daf‐16(*mu86), and *daf‐2*(e1370) mutants. In all three genetic backgrounds, neither compound extended lifespan (Figures S7A–J; Table S7), indicating that both ATFS‐1 and DAF‐16 are necessary for their beneficial effects.

Unlike doxycycline, whose lifespan‐extending effects are largely restricted to ATFS‐1‐dependent UPRmt activation (Houtkooper et al. 2013; Wu et al. 2018), terbinafine and miglustat engage both mitochondrial stress signaling and IIS signaling. This dual requirement suggests a more integrated stress response. This interplay aligns with observations in long‐lived mitochondrial mutants, where both transcription factors are essential for lifespan extension (Senchuk et al. 2018; Wu et al. 2018).

While ATFS‐1 has been shown to promote DAF‐16 nuclear localization, we found that DAF‐16 also supports ATFS‐1 activation under pharmacological stress. Knockdown of *daf‐16* reduced ATFS‐1 levels in animals treated with terbinafine, miglustat, or doxycycline (Figure S8A–D). Specifically, *daf‐16* knockdown led to a 31% decrease in ATFS‐1 levels under doxycycline, a 22% decrease under terbinafine, and a 9% decrease under miglustat, suggesting that DAF‐16 promotes ATFS‐1 expression across multiple contexts. Conversely, *atfs‐1* knockdown impaired DAF‐16 nuclear localization in terbinafine and miglustat treated animals, decreasing to 55% and 19%, respectively (Figure S8E–G), but not under doxycycline treatment (increasing to 63%), indicating stressor‐specific interaction between these two factors.

Finally, we asked whether canonical IIS inhibition is sufficient to activate ATFS‐1. RNA interference of *daf‐2* induced a modest (~2‐fold) increase in nuclear ATFS‐1 (Figure S9A,B). However, *atfs‐1* knockdown did not prevent DAF‐16 nuclear localization under IIS inhibition (Figure S9C), indicating that ATFS‐1 is not required for canonical DAF‐16 activation.

Together, these data support a model in which pharmacological mitochondrial stress engages a bidirectional and context‐dependent regulatory loop between ATFS‐1 and DAF‐16, distinct from canonical IIS signaling.

### 2.6 Terbinafine and Miglustat Induce Mitochondrial Stress and Activate the Integrated Stress Response in Human Cells

**FIGURE 5:**
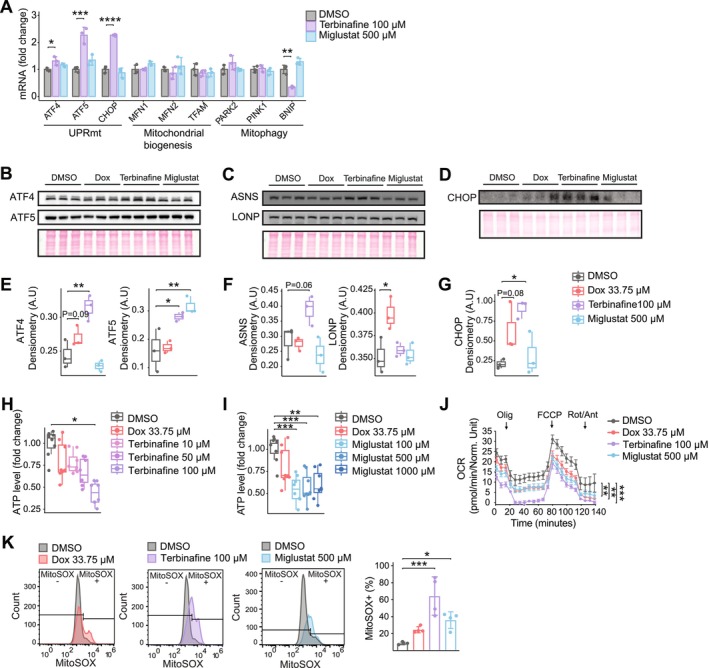
Activation of the MSR and induction of mitochondrial dysfunction by terbinafine and miglustat in HEK293T cells. (A) RT‐qPCR analysis of MSR gene expression (UPRmt, mitophagy, and biogenesis) in HEK293T cells after 24‐h treatment (*n* = 3). (B–G) Western blot analysis and densitometry quantification of mitochondrial stress markers. Statistical analysis was performed using one‐way ANOVA with Bonferroni correction (*p < 0.05, **p < 0.01). (H–I) ATP content in HEK293T cells 24‐h after treatment with various dose of terbinafine (100 μM) (H) and miglustat (500 μM) (I). Results were analyzed by one‐way ANOVA with Bonferroni adjustment (*p < 0.05, **p < 0.01, ***p < 0.001). (J) Oxygen consumption rate (OCR) measurements in HEK293T cells post a 24‐h treatment with DMSO, doxycycline (33.75 μM), terbinafine (100 μM) or miglustat (500 μM). Olig, oligomycin; FCCP, carbonyl cyanide‐p‐trifluoromethoxyphenylhydrazone; Rot/Ant, rotenone/antimycin (*n* > 10). (K) Flow cytometry analysis of MitoSOX fluorescence in HEK293T cells, quantifying an increase in reactive oxygen species (ROS) after a 24‐h exposure to doxycycline (33.75 μM), terbinafine (100 μM) or miglustat (500 μM), relative to control (*n* = 4). Significance was determined using one way ANOVA with Bonferroni correction (*p < 0.05, **p < 0.01, ***p < 0.001).

To evaluate the therapeutic potential of our findings, we investigated the effects of terbinafine and miglustat on mitochondrial and cellular stress responses in human embryonic kidney 293T (HEK293T) cells. RT‐qPCR analysis revealed that terbinafine robustly up‐regulated genes associated with the UPRmt, while miglustat had no significant effect on mitochondrial stress‐related gene expression (Figure 5A). This differential gene expression was reflected at the protein level, where terbinafine induced the expression of key stress response factors ATF4, ATF5, C/EBP homologous protein (CHOP), and the mitochondrial stress protein ASNS (Figure 5B–G). In contrast, miglustat selectively up‐regulated ATF5, with minimal effects on other stress response proteins (Figure 5B–G).

Interestingly, the transcription factors, particularly ATF4 and CHOP, are central mediators of the integrated stress response (ISR) (Costa‐Mattioli and Walter 2020; Quirós et al. 2017), a conserved adaptive pathway activated by various cellular stressors, including mitochondrial dysfunction. Central to the ISR is the phosphorylation of eukaryotic translation initiation factor 2 alpha (eIF2α), which reduces global protein synthesis while selectively promoting the translation of stress response genes. Notably, these factors are also involved in the endoplasmic reticulum (ER) stress response, triggered by the accumulation of misfolded proteins in the ER. To determine whether ER stress contributes to the mechanism of action of terbinafine or miglustat, we examined the expression of the ER‐specific chaperone BiP‐GRP78 (immunoglobulin heavy chain‐binding protein, BiP) (Costa‐Mattioli and Walter 2020; Mottis et al. 2019). Neither compound altered BiP‐GRP78 levels, suggesting that ER stress is not a significant component of their mechanism (Figure S10).

To further characterize the impact of terbinafine and miglustat on mitochondrial function, we measured cellular ATP levels and performed respirometry analysis in HEK293T cells. Both compounds caused a dose‐dependent reduction in ATP levels (Figure 5H,I), significantly decreased basal and maximal respiration rates (Figure 5J), and increased mitochondrial ROS production (Figure 5K). These findings suggest that terbinafine and miglustat induce mitochondrial dysfunction and oxidative stress, consistent with their activation of mitochondrial stress pathways. Overall, our results provide evidence for the conserved action of terbinafine and miglustat as mitochondrial stressors across species, from *C. elegans* to human cells.

### 2.7 Terbinafine Induces a Mitonuclear Protein Imbalance, Similar to the Mechanism Employed by Doxycycline

**FIGURE 6:**
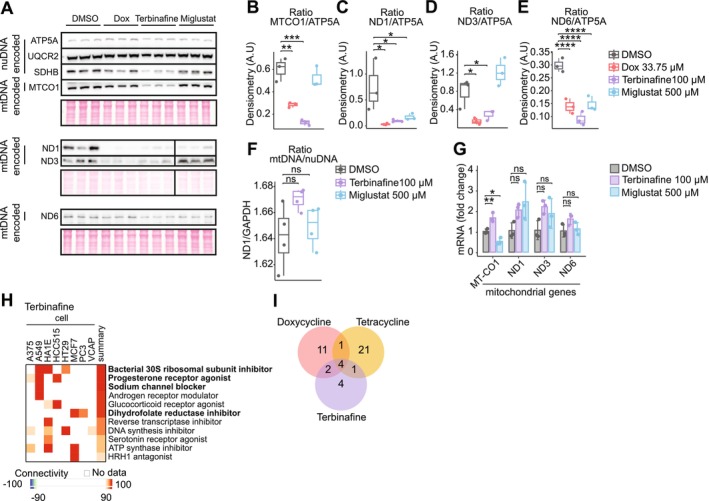
Terbinafine induces a mitonuclear protein imbalance in HEK293T, similarly to doxycycline. (A–E) Western blot analysis and quantification of OXPHOS subunits. terbinafine (100 μM) treatment disrupts the nuclear‐to‐mitochondrial‐encoded OXPHOS ratio. Statistical significance was determined using one‐way ANOVA with Bonferroni adjustment (*p < 0.05, **p < 0.01, ***p < 0.001, ****p < 0.0001). (F) Quantification of mitochondrial content through qPCR analysis of mitochondrial (ND1) and nuclear DNA (GAPDH) in HEK293T cells following a 24‐h treatment (*n* = 4). Statistical analysis was performed using one‐way ANOVA with Bonferroni correction (*p < 0.05, **p < 0.01). (G) RT‐qPCR analysis of mitochondrial gene expression in HEK293T cells after treatment (*n* = 3). Statistical analysis was performed using one‐way ANOVA with Bonferroni correction (*p < 0.05, **p < 0.01). (H) Connectivity map (CMap) analysis revealing similarities between terbinafine and known mitochondrial translation inhibitors. (I) Venn diagram of transcriptional overlap, 63.6% similarity between terbinafine, doxycycline and tetracycline signatures.

We have established the activation of MSR and ISR by terbinafine and miglustat in human cells. Although the effect on mitochondrial translation in worms was subtle, we investigated whether these compounds influence mitochondrial translation, a critical determinant of mitochondrial proteostasis. In HEK293T cells, western blot analysis of oxidative phosphorylation (OXPHOS) complex subunits revealed that terbinafine treatment disrupted the nuclear‐to‐mitochondrial‐encoded (nuDNA/mtDNA‐encoded) OXPHOS protein ratio, indicative of a mitonuclear protein imbalance (Figure 6A–E). This imbalance, a hallmark of MSR activation (Houtkooper et al. 2013), was comparable to that induced by doxycycline, whereas miglustat had a weaker impact. Neither mitochondrial content nor mtDNA‐encoded transcript levels were broadly altered, suggesting that the imbalance reflects impaired mitochondrial translation rather than changes in mitochondrial abundance (Figure 6F,G). We interpret the increase in MT‐CO1 (mitochondrially encoded cytochrome c oxidase subunit 1) mRNA (Figure 6G) levels by terbinafine as a compensatory response to the drastic reduction in MT‐CO1 protein levels (Figure 6A,B).

The similarity between terbinafine and doxycycline is notable, as doxycycline is a well‐characterized mitochondrial translation inhibitor. It disrupts mitochondrial ribosome function, inducing mitonuclear protein imbalance and activating the MSR that extends lifespan in *C. elegans* . Our findings suggest that terbinafine may act through a similar mechanism, potentially targeting mitochondrial translation machinery.

To explore this hypothesis, we conducted a comparative transcriptomic analysis using the Connectivity Map (CMap) database (Subramanian et al. 2017), which includes over 1.5 million gene expression profiles from 5000 small molecules and 3000 genetic perturbations across multiple human cell lines. Terbinafine's gene expression signature showed a 63.6% overlap with those of the established UPRmt inducers, doxycycline and tetracycline (Figure 6H,I). Notably, terbinafine exhibited a striking similarity to the bacterial 30S ribosomal subunit inhibition signature (Figure 6H). These results provide additional evidence that terbinafine, like doxycycline and tetracycline, may extend healthspan and lifespan by inducing a mitonuclear protein imbalance through inhibition of mitochondrial translation.

By disrupting mitonuclear protein homeostasis, terbinafine may activate the MSR, offering a potential mechanism for its healthspan and lifespan‐extending effects.

## 3 Discussion

Aging is a growing global health challenge, with current treatments often targeting symptoms rather than the underlying causes. Here, we identified two FDA‐approved drugs, terbinafine and miglustat, as novel mitochondrial stress modulators that extend both lifespan and healthspan. Although both compounds promote longevity, our data indicate that they engage partially overlapping but mechanistically distinct stress adaptation programs, highlighting the complexity of mitohormetic regulation.

Terbinafine, an antifungal agent targeting squalene epoxidase (SQLE), robustly activated the UPRmt via ATFS‐1 and disrupted mitochondrial translation, similar to doxycycline's mechanism of action. In contrast, miglustat, currently used to treat lysosomal storage disorders, showed a more modest activation of ATFS‐1. Both compounds also activated the insulin/IGF‐1 signaling (IIS) pathway, as evidenced by DAF‐16 nuclear translocation and regulation of IIS target genes. In contrast, doxycycline's effects were restricted to UPRmt activation through ATFS‐1.

Our findings support a functional interaction between the IIS and MSR pathways. While ATFS‐1 has been shown to facilitate DAF‐16 nuclear localization (Wu et al. 2018), we find that DAF‐16 can reciprocally facilitate ATFS‐1 activation under pharmacological stress. This differs from canonical IIS inhibition, in which FOXO/DAF‐16 activation precedes mitochondrial remodeling and does not require ATFS‐1.

Together, these observations suggest that mitohormetic compounds can engage a non‐canonical signaling sequence in which mitochondrial perturbation itself contributes to FOXO activation, revealing a bidirectional and context‐dependent interplay between mitochondrial and IIS pathways, and providing a mechanistic framework for how mitochondrial stress can actively engage IIS/FOXO to promote longevity.

Beyond ATFS‐1 and DAF‐16, transcription factor motif analyses revealed enrichment for additional stress‐responsive regulators, including GATA transcription factors and PQM‐1. Given their established roles in stress resilience and longevity in *C. elegans* (Li et al. 2025), these findings support a model in which mitohormetic lifespan extension relies on a coordinated transcriptional program rather than a single master regulator.

The effects of terbinafine and miglustat in human cells further support their therapeutic potential. In HEK293T cells, terbinafine activated key stress regulators, impaired mitochondrial respiration, and increased ROS production, while miglustat had a more subtle impact, selectively activating ATF5 and mitochondrial function. Comparative transcriptomics revealed strong similarities between terbinafine and known mitochondrial translation inhibitors such as doxycycline, suggesting that terbinafine may act via mechanisms similar to those of doxycycline. These findings were further supported by evidence of a mitonuclear protein imbalance, a hallmark of mitochondrial translation inhibition. Unfortunately, limitations in available data limited our ability to conduct a similar exploration for miglustat.

An important open question is whether the interplay between mitochondrial stress signaling and FOXO activity observed in *C. elegans* is conserved in mammals. While both compounds induced mitochondrial stress responses in human cells, FOXO signaling was not directly assessed. Determining whether mitochondrial perturbation can similarly engage FOXO‐dependent adaptive programs in mammals represents an important direction for future studies.

Finally, our study highlights both the promise and the challenges of identifying mitohormetic compounds. Many initial hits failed to extend lifespan due to toxicity, underscoring the delicate balance required for beneficial stress responses. Moreover, manual dose–response limited a full exploration of hormetic ranges, suggesting that automated dose–response and transcriptomics‐guided approaches could improve precision in future screens. Although no significant toxicity was observed in worms or HEK293T cells at lifespan‐extending concentrations, translation to mammalian models will require careful dose optimization and safety monitoring, particularly given the known side‐effect profiles of terbinafine and miglustat. In addition, while neither compound inhibited the growth of the standard *E. coli* OP50 diet under our experimental conditions, future work should test a broader panel of commensal bacteria to fully exclude unintended antimicrobial effects and potential microbiome perturbation.

In conclusion, the identification of terbinafine and miglustat as novel mitochondrial stress modulators provides a promising opportunity for developing new therapeutic strategies to combat aging. Their FDA‐approved status, combined with the absence of significant antibacterial activity in our assay, positions them as particularly interesting candidates for clinical application. Moreover, our findings regarding the integration of multiple stress response pathways offer new therapeutic directions for combating age‐related diseases and promoting healthy aging.

## 4 Materials and Methods

### 4.1 Chemicals

The Screen‐Well FDA‐approved drug library V2 (https://www.enzolifesciences.com/BML‐2843/screen‐well‐fda‐approved‐drug‐library‐v2/) was purchased from Enzo Life Sciences (Brussels, Belgium). Doxycycline (Dox; Cat. D9891) was obtained from Sigma‐Aldrich (Zwijndrecht, The Netherlands), and terbinafine (BML‐EI318‐0100), and miglustat (BML‐SL230‐0025) were obtained from Enzo Life Sciences.

### 4.2 *C. elegans* Strain and Worm Collection for the Two‐Step Screening Process

Worm strains were provided by the *Caenorhabditis* Genetics Center (University of Minnesota). The strain SJ4100 (*zcIs13[hsp‐6::GFP]*) was used for screening. N2, *atfs‐1*(gk3094), *daf‐16(*mu86), and *daf‐2*(e1370), were used for lifespan assays. OP675 (*atfs‐1p::atfs‐1::FLAG::GFP*), TJ356 (*daf‐16p::daf‐16a/b::GFP*), and SJ4103 (*myo‐3p::mtGFP*), ZG120 (*unc‐119(ed3) III; iaIs7 IV., [nhr‐57p::GFP + unc‐119(+)]*), ZG119 (*unc‐119(ed3) III; iaIs7 IV; vhl‐1(ok161)* X., *[nhr‐57p::GFP + unc‐119(+)]*), CF1553 (*sod‐3p::GFP*), SJ4005 (*hsp‐4::gfp*), CL2070 (*hsp‐16.2p::GFP*), the *mtRosella* biosensor IR2539 [*unc‐119(ed3)*; Ex[*pmyo‐3::TOMM‐20::Rosella; unc‐119(+)*]], was used for imaging‐based assays (Palikaras et al. 2015).

### 4.3 Compound Treatment in *C. elegans*

For the screening, worms were treated from egg hatching to young adult (3 days) on *E. coli* HT115 seeded 12‐well plates. Drugs were administered on the plate's surface (with 2 mM IPTG, 25 mg/mL carbenicillin, 10 μM 5‐FU) at 100 μM or 50 μM if toxicity was observed. Each batch of experiments included a positive control of 50 μM doxycycline and a negative control of 1% DMSO. Regimens were designed to ensure that DMSO concentration did not exceed 1%. For the lifespan assays, compounds were administered from L1 larvae through adulthood. Treatments were supplemented to the plate surface (containing 2 mM IPTG, 25 mg/mL carbenicillin, and 10 μM 5‐FU). Terbinafine (31.5 μM), miglustat (250 μM), and doxycycline (50 μM) were similarly administered for compound characterization.

### 4.4 Lifespan and Healthspan Measurements

Lifespan was carried out at 20°C as described (Mouchiroud et al. 2011), with 60–80 worms per condition scored every other day. Paralysis was assessed as previously described (Leiteritz et al. 2020).

### 4.5 Mitochondrial Stress Screening Image Acquisition and Analysis

Day 1 adult worms were immobilized with 7.5 mM tetramisole hydrochloride (Cat. T1512, Sigma), and imaged using a Nikon SMZ1000 microscope. GFP signals were quantified with FIJI/ImageJ (https://imagej.net/Fiji).

Values were normalized, and *Z*‐scores were calculated as follows:

$$Z_{{\text{score}}_{k}}=\frac{\mu_{k}-\mu_{\text{DMSO}}}{\sigma_{\text{DMSO}}}$$

where *μ*_K_ and *μ*_DMSO_ represent the mean fluorescence intensity in compound‐treated and control worms, respectively. σ_DMSO_ is the standard deviation of the negative control.

### 4.6 GFP Quantification and Confocal Imaging in *C. elegans*

To assess mitochondrial content, we used SJ4103 (zcIs14[*myo‐3p::mtGFP*]) worms. Worms were treated from L1 to day 1 adult, then collected and transferred to 96‐well plates (10–15 animals/well). GFP intensity was measured using a SpectraMax iD3 plate reader (Molecular Devices) and normalized to worm number. For the confocal imaging of OP675 and TJ356, worms were immobilized with 10 mM tetramisole and mounted on 2% agarose pads. Images were captured using a Leica SP8 confocal microscope. Mitophagy was assessed using the *mtRosella* biosensor strain IR2539 (Palikaras et al. 2015). Worms were imaged using a Leica DM5500 fluorescence microscope and mitophagy levels were quantified as the ratio of the pH‐insensitive DsRed signal to the pH‐sensitive GFP signal.

### 4.7 Bacterial Growth Test

*E. coli* OP50 were harvested at 37°C in LB (Luria‐Bertani) medium with compounds. Growth was assessed by measuring optical density at 600 nm (OD 600) every hour for 8 h using a SpectraMax iD3 plate reader.

### 4.8 Worm Sample Collection for RNA Sequencing

L1 larvae (~3000 worms per condition) were treated, collected at Day 1 of adulthood, washed, and snap‐frozen in liquid nitrogen. We collected four biological replicates per condition.

### 4.9 *C. elegans* RNA Extraction and RNA‐Seq Data Analysis

RNA was extracted using TriPure Isolation Reagent (Cat. 11667165001, Roche). Samples were then freeze‐thawed eight times to disrupt cell membranes. RNA was then extracted using a column‐based RNA extraction kit (Macherey‐Nagel kit, Cat. 740955.250), and sequencing was performed at Beijing Genomics Institute (BGI) on the BGISEQ‐500 platform. The mapping quality was checked using FastQC (fastqcr, version 0.11.7), and reads were aligned to the “ *C. elegans* WBcel235.89” genome using STAR (v2.73a). Differential gene expression analysis was conducted with DEseq2 (v1.40.2) (Love et al. 2014). Gene Set Enrichment Analysis (GSEA) was performed using clusterProfiler (v42.2) (Yu et al. 2012). The enrichment used a minimum gene set size of 1, a maximum gene set size of 5000, and performed 100,000 permutations.

### 4.10 Transcription Factor Enrichment Analysis

Motif enrichment analysis was performed using HOMER (v4.11) (Duttke et al. 2019) on differentially expressed genes (abs(log_2_FC) > 0.5, adjusted *p*‐value < 0.05). De novo motif discovery was performed with *findMotifs.pl* (start: −500 bp; end: 500 bp) against the library of known motifs from HOMER and data downloaded from the JASPAR database (https://jaspar2020.genereg.net/).

### 4.11 Mitochondrial Oxygen Consumption, ATP, ROS Measurement in *C. elegans*

Oxygen consumption rate (OCR) was measured using a Seahorse XF96 (Agilent Technologies). 100–150 L1 worms were treated, collected on Day 1 of adulthood, washed and transferred to the Seahorse plates (5–10 worms per well). Maximal OCR was assessed after 10 μM FCCP injections, and values were normalized to worm count. Mitochondrial reactive oxygen species (mtROS) levels were assessed by staining animals with MitoTracker Red CM‐H_2_XROS (Molecular Probes, Invitrogen; M7513), while mitochondrial ATP levels were measured using the BioTracker ATP‐Red live‐cell dye (Merck; SCT045). Both dyes were diluted in M9 buffer and added to the surface of pre‐seeded nematode growth medium (NGM) plates at final concentrations of 0.15 μM (MitoTracker Red CM‐H_2_XROS) and 0.8 μM (BioTracker ATP‐Red).

### 4.12 Cell Culture and Drug Treatment

HEK293T cells (Cat. CRL‐3216, ATCC) were cultured in DMEM (4.5 g/L glucose, 10% FBS) at 37°C with 5% CO_2_. Cells were treated with doxycycline (33.75 μM—15 μg/mL), terbinafine (100 μM), or miglustat (500 μM) for 24 h.

### 4.13 Cell RNA Extraction and RT‐qPCR

Total RNA was extracted with 1 mL of TriPure Isolation Reagent (Cat. 11667165001, Roche) and purified using the Macherey‐Nagel RNA kit. cDNA was synthesized using the Qiagen Reverse Transcription Kit (Cat. 205314) and RT‐qPCR was performed with LightCycler 480 SYBR Green I Master kit (Cat. 04887352001, Roche). The primer sequences are provided in Supporting Information (Table S8). Expression values were normalized to GAPDH.

### 4.14 Mitochondrial Respiration and ATP Measurement in HEK293T Cells

OCR was measured using a Seahorse analyzer XF96, assessing basal, maximal respiration, and non‐mitochondrial respiration (2 μM Oligomycin, 2 μM FCCP, 1 μM Rotenone/Antimycin). OCR values were normalized to DNA content (Hoechst staining). ATP levels were quantified using CellTiter‐Glo (Promega), with luminescence recorded using SpectraMax iD3 and normalized to protein content (Lowry assay).

### 4.15 Mitochondrial ROS Quantification in HEK293T Cells

Mitochondrial ROS levels were measured using MitoSox (Cat. M36008, Thermofisher). Cells were incubated with 1 μM MitoSox at 37°C for 30 min, washed twice, detached using cold PBS, and analyzed by flow cytometry (FlowJo LLC).

### 4.16 Western Blot in HEK293T Cells

The Western blot was conducted as previously described (Benegiamo et al. 2023). Cells were lysed using RIPA buffer (50 mM Tris–HCl pH 7.4, 150 mM NaCl, 1% NP‐40, 0.5% Na‐deoxycholate, 0.1% SDS, 2 mM EDTA, and 50 mM NaF) with protease and phosphatase inhibitor cocktails (Roche/Thermo Fisher Scientific). Proteins were quantified by the Lowry assay. Gel electrophoresis was run at 200 V for 1 h in 3‐(N‐morpholino) propanesulfonic acid–SDS running buffer. Proteins were then transferred to nitrocellulose membranes (100 V, 2 h, on ice). Blocking was performed in 5% milk/TBST for 1 h, and incubated overnight at 4°C with primary antibodies (see Supporting Information, Table S9). Following three washes with a solution of Tris‐buffered saline and Tween 20, membranes were incubated with HRP‐conjugated anti‐mouse, anti‐rabbit, anti‐goat secondary antibody, depending on the antibody used. Images were quantified using FIJI/ImageJ and normalized to Ponceau staining.

## Author Contributions

Conceptualization: Amélia Lalou, Arwen W. Gao, Johan Auwerx. Data analysis: Amélia Lalou. Methodology: Amélia Lalou, Sandra Rodríguez‐López, Arwen W. Gao, Terytty Yang Li, Qi Wang, Investigation: Amélia Lalou. Assistance with investigation: Ioanna Daskalaki, Ilias Gkikas, Sandra Rodríguez‐López, Adrien Faure, Joaquim Barmaz, Arwen W. Gao, Feng Gao, Danaé Broustail. Writing – original draft: Amélia Lalou, Jean‐David Morel, Giorgia Benegiamo, Johan Auwerx. Writing – reviewing and editing: all authors. Funding acquisition: Johan Auwerx, Kristina Schoonjans, Giovanni D'Angelo, Supervision: Johan Auwerx.

## Funding

We acknowledge funding from the Ecole Polytechnique Fédérale de Lausanne (J.A.), the European Research Council (ERC‐AdG‐787702), the Swiss National Science Foundation (SNSF 31003A_179435 and Sinergia CRSII5_202302), and a GRL grant of the National Research Foundation of Korea (NRF 2017K1A1A2013124). S.R.‐L. was supported by the European Molecular Biology Organization (ALTF 67‐2022). T.Y.L. was supported by the “Human Frontier Science Program” (LT000731/2018‐L). Q.W. was supported by EMBO (ALTF 111‐2021). A.W.G. was supported by the United Mitochondrial Disease Foundation (PF‐19‐0232), Amsterdam University Medical Centers (Amsterdam UMC) through the Postdoc Career Bridging Grant, the European Commission through the Horizon Europe programme (MSCA‐PF‐EF‐2022, 101108082), and Amsterdam UMC through the AGEM Talent Development Grant (2023).

## Conflicts of Interest

The authors declare no conflicts of interest.

## Supporting information


**Figure S1:** Dose–response analysis of the top 18 compounds in *hsp‐6p::gfp* worms.
*hsp‐6p::gfp* reporter strain was raised on NGM supplemented by vehicle (1% DMSO) or various concentrations of the compounds (31.25, 62.5, 125, 250, 500, and 1000 μM). Empty panels represent toxic conditions. Drug names in blue indicate selected compounds and arrows indicate the concentrations selected for further analysis. Scale bars, 0.3 mm.
**Figure S2:** Lifespan measurements of worms treated with the selected compounds. *x*‐axis shows lifespan in days. *y*‐axis shows the percentage of worms alive. Statistics are reported in Table S2.
**Figure S3:** Evaluating the impact of terbinafine and miglustat on development and reproductive capacity. (A) Egg‐laying rate of adult worms following doxycycline (50 μM), terbinafine (31.25 μM), miglustat (250 μM) treatment. Reproductive capacity is measured by the number of eggs laid by animal per hour (No. of eggs/animal/h). Significance was determined using two‐way ANOVA with Tukey correction (*p < 0.05, **p < 0.01, ***p < 0.001). (B, C) Worm body size at Day 1 adult stage following various concentrations of terbinafine (B) and miglustat (C). Body size is characterized by the perimeter (mm) and the area (mm^2^), and the animal morphology by the area‐to‐perimeter ratio. Significance was determined using one way ANOVA with Bonferroni correction (*p < 0.05, **p < 0.01, ***p < 0.001).
**Figure S4:** Evaluating the impact of terbinafine and miglustat on mitophagy. (A) Heatmap of the expression of mitophagy related‐genes following treatment with doxycycline (50 μM), terbinafine (31.25 μM), miglustat (250 μM) versus control. Expression values are represented in log_2_ fold changes (log_2_FC). Statistical significance after FDR correction for multiple testing are indicated as follow: *p < 0.1, **p < 0.05, ***p < 0.01. (B) Quantitative RT‐PCR analysis of mitochondrial biogenesis, mitophagy and autophagy related‐genes, in *C. elegans* following treatment with terbinafine (15 μM, 31.25 μM) or miglustat (250 μM, 500 μM). Significance was determined using one way ANOVA with Bonferroni correction (*p < 0.05, **p < 0.01, ***p < 0.001). (C, D) Representative confocal images of mtRosella biosensor worms (C). Mitophagy levels were quantified by calculating the ratio of the pH‐insensitive DsRed signal to the pH‐sensitive GFP signal, as previously described. An increase in this ratio reflects enhanced delivery of mitochondria to acidic lysosomal compartments indicating enhanced mitophagy rates (D). Significance was determined using one way ANOVA with Bonferroni adjustment (*p < 0.05, **p < 0.01, ***p < 0.001).
**Figure S5:** Enriched transcription factor motifs and interaction networks following treatment with doxycycline, terbinafine, and miglustat. (A) Presentation of all the transcription factor motifs enriched in the promoters of genes up‐regulated by doxycycline (Dox) (50 μM), terbinafine (31.25 μM), and miglustat (250 μM), as analyzed by HOMER. The column ‘Best match’ signifies the transcription factor that most closely corresponds to the enriched motif. Adjusted *p*‐values are provided for each motif. (B–D) Protein–protein interaction networks generated by STRING, using the enriched transcription factors identified for each treatment. Colored nodes highlight key transcription factors involved in various cellular stress responses, while gray nodes represent interacting proteins. These networks provide insight into the complex regulatory mechanisms activated by doxycycline, terbinafine, and miglustat.
**Figure S6:** Terbinafine and miglustat treatment effect on multiple stress response pathways. (A) The *hsp‐16.2p::GFP* reporter strain was cultivated on NGM supplemented with compounds and fed with *E. coli* HT115, illustrating heat shock stress activation. Under basal conditions, neither terbinafine nor miglustat induced the heat shock response. Upon heat stress, worms treated with both compounds displayed an enhanced induction of *hsp‐16.2p::GFP*, as indicated by increased GFP fluorescence, indicating that these compounds do not activate the heat shock response but can potentiate heat shock responsiveness under stress conditions. (B) The *nhr‐57p::GFP* (ZG120, ZG119) grown under the same conditions, monitoring hypoxia‐responsive transcription. The ZG119 strain carries a *vhl‐1* mutation, resulting in constitutive activation of the hypoxia pathway and serving as a positive control. (C) The *sod‐3p::GFP* reporter strain was cultivated on NGM supplemented with compounds and fed with *E. coli* HT115, illustrating oxidative stress activation. (D) The *hsp‐4p::GFP* reporter strain was cultivated on NGM supplemented with compounds and fed with *E. coli* HT115, illustrating ER stress activation.
**Figure S7:** Lifespan measurements of the *atfs‐1*(gk3094), *daf‐16*(mu86) and *daf‐2*(e1370) mutants treated with terbinafine and miglustat. *x*‐axis shows lifespan in days. *y*‐axis shows the percentage of worms alive. Statistics are reported in Table S7.
**Figure S8:** ATFS‐1 and DAF‐16 regulatory interplay under pharmacological interventions. (A) Microscopic images of intestinal cells in *atfs‐1p::atfs‐1::flag::gfp* worms treated with doxycycline (Dox) (50 μM), terbinafine (31.25 μM), and miglustat (250 μM) or vehicle along with or without *daf‐16* RNAi. Nuclei are outlined with white dashed circles, and autofluorescent lysosomes appear as punctate structures. (Figure S8B‐D) Quantification of the fluorescence intensity in the nucleus and cytoplasm of intestinal cells, highlighting ATFS‐1 expression and localization (*n* > 10 biological replicates). Statistical significance was assessed using one‐way ANOVA with Bonferroni correction for multiple comparisons (*p < 0.05, **p < 0.01, ***p < 0.001, ****p < 0.0001). (E–G) DAF‐16 localization is classified as cytoplasmic, intermediate or nuclear. The histogram represent the proportion of the number of worms with cytosolic, intermediate, or nuclear localization of DAF‐16 following treatment with doxycycline (Dox) (50 μM), terbinafine (31.25 μM), and miglustat (250 μM) or vehicle along with or without *atfs‐1* RNAi (*n* ≥ 8).
**Figure S9:** *atfs‐1* is not required for DAF‐16 activation under reduced IIS signaling. (A) Microscopic images of intestinal cells in *atfs‐1p::atfs‐1::flag::gfp* worms treated with control RNAi, *daf‐2* RNAi, with or without concurrent *daf‐16* RNAi (50%). Nuclei are outlined with white dashed circles, and autofluorescent lysosomes appear as punctate structures. (B) Quantification of the fluorescence intensity in the nucleus and cytoplasm of intestinal cells, highlighting ATFS‐1 expression and localization (*n* > 10 biological replicates). Statistical significance was assessed using one‐way ANOVA with Bonferroni correction for multiple comparisons (*p < 0.05, **p < 0.01, ***p < 0.001, ****p < 0.0001). (C) DAF‐16 localization is classified as cytoplasmic, intermediate or nuclear. The histogram represent the proportion of worms with cytosolic, intermediate, or nuclear localization of DAF‐16 following control RNAi, *daf‐2 *RNAi, with or without concurrent *atfs‐1* RNAi (50%) (*n* ≥ 8).
**Figure S10:** Terbinafine and miglustat do not induce ER Stress as evidenced by BiP‐GRP78 expression levels. (A) Quantitative RT‐PCR analysis of BiP‐GRP78 gene expression in HEK293T cells following 24‐h treatment with terbinafine (100 μM) or miglustat (500 μM), indicating reduced expression in miglustat and no change in terbinafine (*n* = 3 biological replicates). (B, C) Western blot analysis and corresponding densitometry quantification demonstrate BiP‐GRP78 protein expression changes post 24‐h treatment with terbinafine or miglustat, indicating no significant change in BiP‐GRP78 expression following treatments (*n* = 3 biological replicates). Statistical analysis was performed using one‐way ANOVA with Bonferroni correction for multiple comparisons, where *p < 0.05, **p < 0.01 signify significant differences.

**Table S1:** Description of the 20 most promising mitochondrial stress modulators.
This table presents the 20 leading candidates identified. For each compound, the table details its name, indication, drug classification (KEGG drug database), target and mechanism (Drug Bank database). This table underscores the vast variety of mechanism of action and therapeutic applications of these compounds.
**Table S2:** Summary of lifespan experiments.
Summary of mean and median lifespans and statistical analysis (*p*‐values) for lifespan experiments. *p*‐values are from a log rank test comparing treated population to the control. *p*‐values less than 0.05 are considered statistically significant. The total number of individuals per experiment is shown (NTOTAL).
**Table S3:** List of the 11 genes similarly up‐regulated in the three treatments.
Description of the genes specifically up‐regulated in response to terbinafine, miglustat and doxycycline treatment, along with their associated Gene Ontology (GO) categories.
**Table S4:** List of the 37 genes exclusively up‐regulated by terbinafine.
Description of the genes specifically up‐regulated in response to terbinafine treatment, along with their associated Gene Ontology (GO) categories. N.A. indicates that no annotation is currently available in WormBase (Version: WS295) for the respective gene.
**Table S5:** List of the 18 genes exclusively up‐regulated by miglustat.
Description of the genes specifically up‐regulated in response to miglustat treatment, along with their associated Gene Ontology (GO) categories. N.A. indicates that no annotation is currently available in WormBase (Version: WS295) for the respective gene.
**Table S6:** List of the 3 genes up‐regulated by both terbinafine and miglustat.
Description of the genes commonly up‐regulated by terbinafine and miglustat but not by doxycycline, along with their associated Gene Ontology (GO) categories.
**Table S7:** Summary of lifespan experiments of the *atfs‐1*(gk3094), *daf‐16*(mu86) and *daf‐2*(e1370) mutants.
Summary of mean and median lifespans and statistical analysis (*p*‐values) for lifespan experiments. *p*‐values are from a log rank test comparing treated population to the vector control. *p*‐values less than 0.05 are considered statistically significant. The total number of individuals per experiment is shown (NTOTAL).
**Table S8:** List of PCR primers.
This table details the sequences of the primers used, as described in the Materials and Methods section. For each gene, the table provides the forward (Fw) and reverse (Rv) primer sequence (5′ to 3′ orientation).
**Table S9:** List of antibodies.
This tables details the antibodies used for Western blotting, as described in the Materials and Methods section. For each antibody, the target protein, reference number and manufacturer are provided.

## Data Availability

The data that support the findings of this study are openly available in NCBI Gene Expression Omnibus at https://www.ncbi.nlm.nih.gov/geo/, reference number GSE290712.
