# Supplementary material for: Targeting Mitochondrial Stress Responses: Terbinafine and Miglustat as Novel Lifespan and Healthspan Modulators
Source: Aging Cell. 2026 Mar 30;25(4):e70452. doi: 10.1111/acel.70452 (PMC13092511; doi:10.1111/acel.70452)
Supplement: Supplementary file 2 — Table S1: Description of the 20 most promising mitochondrial stress modulators. This table presents the 20 leading candidates identified. For each compound, the table details its name, indication, drug classification (KEGG drug database), target and mechanism (Drug Bank database). This table underscores the vast variety of mechanism of action and therapeutic applications of these compounds. Table S2: Summary of lifespan experiments. Summary of mean and median lifespans and statistical analysis (p‐values) for lifespan experiments. p‐values are from a log rank test comparing treated population to the control. p‐values less than 0.05 are considered statistically significant. The total number of individuals per experiment is shown (NTOTAL). Table S3: List of the 11 genes similarly up‐regulated in the three treatments. Description of the genes specifically up‐regulated in response to terbinafine, miglustat and doxycycline treatment, along with their associated Gene Ontology (GO) categories. Table S4: List of the 37 genes exclusively up‐regulated by terbinafine. Description of the genes specifically up‐regulated in response to terbinafine treatment, along with their associated Gene Ontology (GO) categories. N.A. indicates that no annotation is currently available in WormBase (Version: WS295) for the respective gene. Table S5: List of the 18 genes exclusively up‐regulated by miglustat. Description of the genes specifically up‐regulated in response to miglustat treatment, along with their associated Gene Ontology (GO) categories. N.A. indicates that no annotation is currently available in WormBase (Version: WS295) for the respective gene. Table S6: List of the 3 genes up‐regulated by both terbinafine and miglustat. Description of the genes commonly up‐regulated by terbinafine and miglustat but not by doxycycline, along with their associated Gene Ontology (GO) categories. Table S7: Summary of lifespan experiments of the atfs‐1(gk3094), daf‐16(mu86) and daf‐2(e1370) mutants. [file ACEL-25-e70452-s002.docx]

**Targeting mitochondrial stress responses: Terbinafine and Miglustat as novel lifespan and healthspan modulators**

Tables

**Supplementary table legends**

**Table S1: Description of the 20 most promising mitochondrial stress modulators.**

This table presents the 20 leading candidates identified. For each compound, the table details its name, indication, drug classification (KEGG drug database), target and mechanism (Drug Bank database). This table underscores the vast variety of mechanism of action and therapeutic applications of these compounds.

**Table S2: Summary of lifespan experiments.**

Summary of mean and median lifespans and statistical analysis (P-values) for lifespan experiments. P-values are from a log rank test comparing treated population to the vector control. P-values less than 0.05 are considered statistically significant. The total number of individuals per experiment is shown (NTOTAL).

**Table S3: List of the 11 genes similarly up-regulated in the three treatments.**

Description of the genes specifically up-regulated in response to Terbinafine, Miglustat and Doxycycline treatment, along with their associated Gene Ontology (GO) categories.

**Table S4: List of the 37 genes exclusively up-regulated by Terbinafine.**

Description of the genes specifically up-regulated in response to Terbinafine treatment, along with their associated Gene Ontology (GO) categories. N.A. indicates that no annotation is currently available in WormBase (Version: WS295) for the respective gene.

**Table S5: List of the 18 genes exclusively up-regulated by Miglustat.**

Description of the genes specifically up-regulated in response to Miglustat treatment, along with their associated Gene Ontology (GO) categories. N.A. indicates that no annotation is currently available in WormBase (Version: WS295) for the respective gene.

**Table S6: List of the 3 genes up-regulated by both Terbinafine and Miglustat.**

Description of the genes commonly up-regulated by Terbinafine and Miglustat but not by Doxycycline, along with their associated Gene Ontology (GO) categories.

**Table S7: Summary of lifespan experiments of the *atfs-1*(gk3094), *daf-16*(mu86) and *daf-2*(e1370) mutants.**

Summary of mean and median lifespans and statistical analysis (P-values) for lifespan experiments. P-values are from a log rank test comparing treated population to the vector control. P-values less than 0.05 are considered statistically significant. The total number of individuals per experiment is shown (NTOTAL).

**Table S8: List of PCR primers.**

This table details the sequences of the primers used, as described in the Materials and Methods section. For each gene, the table provides the forward (Fw) and reverse (Rv) primer sequence (5’ to 3’ orientation).

**Table S9: List of antibodies.**

This tables details the antibodies used for Western blotting, as described in the Materials and Methods section. For each antibody, the target protein, reference number and manufacturer are provided.

| **Common name** | **Disease** | **Drug Class** | **Target** | **Effect** |
| --- | --- | --- | --- | --- |
| Auranofin | Inflammatory arthritis (e.g.rheumatoid arthritis) | Anti-inflammatory | Kappa ß Kinase(IKBKB) | Inhibitor |
|  |  |  | Thioredoxin reductase (PRDX5) | Inhibitor |
| Clotrimazole | Dermatophyte infections and candidiasis | Antifungal | Lanosterol 14-alpha demethylase (ERG11), | Inhibitor |
|  |  |  | Intermediate conductance calcium-activated potassium channel protein 4 (KCNN4) | Inhibitor |
| Leflunomide | Juvenile Idiopathic Arthritis (JIA), Rheumatoid Arthritis | Anti-inflammatory | Dihydroororate deshydrogenase (DHODH) | Inhibitor |
| Minocycline | Infections | Antibacterial | 30S ribosomal protein S9 (rpsI) | Inhibitor |
|  |  |  | 30S ribosomal protein S4 (rpsD) | Inhibitor |
| Flutamide | Prostate cancer | Antineoplastic | Androgen receptor (AR) | Antagonist |
| Miglustat | Gaucher disease | Enzyme Inhibitor | Ceramide glucosyltransferase (UGCG) | Inhibitor |
| Ketoconazole | Fungal infections, Cushing's syndrome | Antifungal | Lanosterol 14-alpha demethylase (ERG11) | Inhibitor |
|  |  |  | Steroid 17-alpha-hydroxylase/17,20 lyase (CYP17A1) | Inhibitor |
| Tetracycline | Infections | Antibacterial | 30S ribosomal protein S7 (rpsG) | Inhibitor |
|  |  |  | 30S ribosomal protein S19 (rpsS) | Inhibitor |
|  |  |  | 30S ribosomal protein S14 (rpsN) | Inhibitor |
|  |  |  | 30S ribosomal protein S8 (rpsH) | Inhibitor |
|  |  |  | 30S ribosomal protein S3 (rpsC) | Inhibitor |
| Silver Sulfadiazine | Wound sepsis | Antibacterial | Toxoplasma gondii, Escherichia coli, Serine/threonine-protein kinase Aurora-A (aurka) | Inhibitor |
|  |  |  | Bacterial dihydropteroate synthase (dhps) | Inhibitor |
| Duloxetine | Anxiety disorder, Neuropathic pain, Osteoarthritis, Stress incontinence | Neuropsychiatric agent | Sodium-dependent serotonin transporter (SLC6A4) | Inhibitor |
|  |  |  | Sodium-dependent noradrenaline transporter (SLC6A2) | Inhibitor |
| Hydrocortisone Acetate | Inflammatory and pruritic corticosteroid-responsive dermatoses and ulcerative colitis | Anti-inflammatory | Glucocorticcoid receptor (NR3C1) | Agonist |
| Clemastine Fumarate | Allergic rhinitis | Anti-allergic agent | H1 receptor (HRH1) | Antagonist |
| Sunitinib Malate | Renal cell carcinoma (RCC) and imatinib-resistant gastrointestinal stromal tumor (GIST) | Antineoplastic | Vascular endothelial growth factor receptor 3 (FLT4) | Inhibitor |
|  |  |  | Platelet-derived growth factor receptor alpha (PDGFRA) | Inhibitor |
|  |  |  | Vascular endothelial growth factor receptor 2 (KDR) | Inhibitor |
|  |  |  | Receptor-type tyrosine-protein kinase FLT3 (FLT3) | Inhibitor |
|  |  |  | Macrophage colony-stimulating factor 1 receptor (CSF1R) | Inhibitor |
|  |  |  | Vascular endothelial growth factor receptor 1 (FLT1) | Inhibitor |
|  |  |  | Stem cell growth factor receptor (KIT) | Inhibitor |
|  |  |  | Platelet-derived growth factor receptor beta (PDGFRB) | Inhibitor |
|  |  |  | Prostaglandin E2 receptor EP4 subtype (PTGER4) | Agonist |
| Alprostadil | Erectile dysfunction | Hormonal agent | Prostanoid EP1 receptor (PTGER1) | Agonist |
|  |  |  | Prostaglandin E2 receptor EP2 subtype (PTGER2) | Agonist |
|  |  |  | Prostaglandin E2 receptor EP3 subtype (PTGER3) | Agonist |
| Fluconazole | Fungal infections including candidiasis | Antifungal | Cytochrome P450 51 | Inhibitor |
| Dextromethorphan | Dry cough | Neuropsychiatric agent | NMDA receptor subunit 3A (GRIN3A) | Antagonist |
|  |  |  | Sigma opioid receptor (SIGMAR1) | Agonist |
| Terbinafine | Fungal skin infections | Metabolizing enzyme substrate | Fungus squalene monooxygenase (erg1) | Inhibitor |
| Bosentan | Pulmonary arterial hypertension | Cardiovascular agent | Endothelin receptor ET-B (EDNRB) | Antagonist |
|  |  |  | Endothelin receptor ET-A (EDNRA) | Antagonist |
| Levonorgestrel | Contraceptives | Hormonal agent | 3-oxo-5-alpha-steroid 4-dehydrogenase 1 (SRD5A1) | Inhibitor |
|  |  |  | Progesterone receptor (PGR) | Modulator |
| Desloratadine | Allergic rhinitis, pruritus, and urticaria | Anti-allergic agent | H1 receptor (HRH1) | Antagonist |

**Table S1**

| Batch | Treatment | Median lifespan (days) | Mean lifespan ± SE (days) | | | Variation compared to control (%) | *P*-values against control | *P*-values summary | NTOTAL |
| --- | --- | --- | --- | --- | --- | --- | --- | --- | --- |
| 1 | DMSO | 22 | 23.589 | ± | 0.481 | 0 |  |  | 58 |
|  | Flutamide 250 µM | 22 | 19.622 | ± | 0.428 | -16.8 | <0.0001 | **** | 76 |
|  | Flutamide 500 µM | 22 | 20.339 | ± | 0.346 | -13.8 | <0.0001 | **** | 79 |
|  | Ketoconazole 500 µM | 22 | 21.152 | ± | 0.376 | -10.3 | <0.0001 | **** | 80 |
|  | Ketoconazole 250 µM | 22 | 21.952 | ± | 0.458 | -6.9 | 0.0077 | ** | 72 |
|  | Miglustat 250 µM | 27 | 24.889 | ± | 0.708 | 5.5 | 0.0153 | * | 74 |
|  | Miglustat 500 µM | 24 | 24.196 | ± | 0.584 | 2.6 | 0.2156 | ns | 70 |
| 2 | DMSO | 28 | 25.721 | ± | 0.738 | 0 |  |  | 79 |
|  | Leflunomide 250 µM | 22 | 19.691 | ± | 0.877 | -23.4 | <0.0001 | **** | 29 |
|  | Leflunomide 500 µM | 16 | 16.740 | ± | 0.165 | -34.9 | <0.0001 | **** | 107 |
|  | Auranofin 125 µM | 14 | 14.418 | ± | 0.320 | -43.9 | <0.0001 | **** | 103 |
|  | Auranofin 250 µM | 14 | 14.412 | ± | 0.359 | -44.0 | <0.0001 | **** | 91 |
| 3 | DMSO | 24 | 25.271 | ± | 0.511 | 0 |  |  | 56 |
|  | Sunitinib malate 31.25 µM | 27 | 25.874 | ± | 0.430 | 2.4 | 0.2885 | ns | 63 |
|  | Sunitinib malate 62.5 µM | 27 | 24.983 | ± | 0.458 | -1.1 | 0.8821 | ns | 64 |
|  | Hydrocortisone acetate 250 µM | 27 | 25.059 | ± | 0.302 | -0.8 | 0.9812 | ns | 55 |
|  | Hydrocortisone acetate 500 µM | 24 | 23.944 | ± | 0.350 | -5.2 | 0.0331 | * | 86 |
|  | Duloxetine 62.5 µM | 17 | 18.362 | ± | 0.447 | -27.3 | <0.0001 | **** | 61 |
|  | Duloxetine 125 µM | 17 | 18.050 | ± | 0.355 | -28.6 | <0.0001 | **** | 64 |
|  | Silver sulfadiazine 62.5 µM | 27 | 24.670 | ± | 0.813 | -2.4 | 0.7686 | ns | 54 |
|  | Silver sulfadiazine 125 µM | 11 | 13.796 | ± | 1.169 | -45.4 | <0.0001 | **** | 41 |
| 4 | DMSO | 27 | 25.238 | ± | 0.548 | 0 |  |  | 66 |
|  | Dextromethorphan 125 µM | 27 | 25.243 | ± | 0.441 | 0 | 0.433 | ns | 70 |
|  | Dextromethorphan 250 µM | 27 | 25.185 | ± | 0.452 | -0.2 | 0.2141 | ns | 58 |
| 5 | DMSO | 26 | 25.657 | ± | 0.439 | 0 |  |  | 72 |
|  | Terbinafine 15 µM | 28 | 27.524 | ± | 0.436 | 7.3 | 0.0009 | *** | 95 |
|  | Terbinafine 31.25 µM | 28 | 28.014 | ± | 0.471 | 9.2 | 0.0001 | *** | 72 |
|  | Clemastine fumarate 15 µM | 26 | 25.907 | ± | 0.428 | 1.0 | 0.6458 | ns | 75 |
|  | Clemastine fumarate 31.25 µM | 24 | 23.952 | ± | 0.558 | -6.6 | 0.0642 | ns | 68 |
|  | Desloratadine 31.25 µM | 26 | 25.452 | ± | 0.510 | -0.8 | 0.6229 | ns | 75 |
|  | Desloratadine 62.5 µM | 26 | 26.386 | ± | 0.560 | 2.8 | 0.1978 | ns | 57 |

**Table S2**

| **Gene** | **Gene Ontology Association** |
| --- | --- |
| *acox-3* | Acyl-CoA oxidase activity, ATP binding, Fatty acid beta-oxidation using acyl-CoA oxidase, Fatty acid beta-oxidation, |
|  | Fatty acid metabolic process, Lipid homeostasis, Lipid metabolic process, Oxidoreductase activity, Peroxisome |
| *hacl-1* | Catalytic activity, Fatty acid alpha-oxidation, Lyase activity, Peroxisome |
| *dhrs-4* | Carbonyl reductase (NADPH) activity, Oxidoreductase activity |
| *F58A6.1* | Delta(3,5)-delta(2,4)-dienoyl-CoA isomerase activity, Fatty acid beta-oxidation, Mitochondrion |
| *dhs-28* | Acyl-CoA metabolic process, Dauer entry, Negative regulation of lipid storage, Oxidoreductase activity, Peroxisome, |
|  | Positive regulation of developmental growth, Very long-chain fatty acid metabolic process |
| *lonp-2* | ATP binding, ATP hydrolysis activity, ATP-dependent peptidase activity, Peroxisome, Protein catabolic process, |
|  | Protein quality control for misfolded or incompletely synthesized proteins, Protein targeting to peroxisome, Proteolysis" |
| *acds-10* | Acyl-CoA dehydrogenase activity, Fatty acid beta-oxidation using acyl-CoA dehydrogenase |
| *daf-22* | Acyl-CoA metabolic process, Acyltransferase activity, Dauer entry, Dauer exit, Lipid binding, Lipid transport, |
|  | Negative regulation of lipid storage, Peroxisome, Positive regulation of developmental growth, |
|  | Propanoyl-CoA C-acyltransferase activity, Very long-chain fatty acid metabolic process |
| *acs-12* | CoA-ligase activity, Ligase activity |
| *maoc-1* | (NAD+) activity, 3-hydroxyacyl-CoA dehydrogenase activity, Defense response to Gram-negative bacterium, |
|  | Enoyl-CoA hydratase activity, Fatty acid beta-oxidation, Innate immune response, Peroxisome |
| *C10C5.4* | Amino acid metabolic process, Aminoacylase activity |

**Table S3**

| **Gene** | **Gene Ontology Association** |
| --- | --- |
| *skpo-2* | Cellular oxidant detoxification, Peroxidase activity, Response to oxidative stress, Collagen-containing extracellular matrix, Heme binding, Metal ion binding |
| *cror-1* | Oxidoreductase activity |
| *sodh-2* | Alcohol dehydrogenase (NAD+) activity, Oxidoreductase activity, Cytoplasm, Metal ion binding |
| *Y53G8B.2* | Acyltransferase activity, Triglyceride biosynthetic process, Diacylglycerol O-acyltransferase activity, Endoplasmic reticulum membrane |
| *dpf-6* | Aminopeptidase activity, Plasma membrane, Proteolysis, Serine-type endopeptidase activity |
| *W01A11.1* | Catabolic process, Catalytic activity, Endoplasmic reticulum membrane, Epoxide hydrolase activity, Epoxide metabolic process, Ether hydrolase activity, Hydrolase activity, Cis-stilbene-oxide hydrolase activity |
| *cpr-4* | Proteolysis, Cellular response to gamma radiation, Cellular response to UV, Cysteine-type endopeptidase activity, Extracellular region |
| *K05B2.4* | Acyl-CoA metabolic process, Fatty acid metabolic process, Fatty acyl-CoA hydrolase activity, Thiolester hydrolase activity |
| *aex-4* | Exocytosis, Positive regulation of protein secretion, Presynapse, SNAP receptor activity, SNARE complex, Synaptic vesicle fusion to presynaptic active zone membrane, Synaptic vesicle priming, Syntaxin binding, Plasma membrane, Positive regulation of defecation |
| *gba-2* | Glucosylceramidase activity, Glucosylceramide catabolic process, Sphingolipid metabolic process, Hydrolase activity |
| *F10D2.8* | Glucuronosyltransferase activity, Glycosyltransferase activity, Membrane, UDP-glycosyltransferase activity |
| *nhr-138* | DNA-binding transcription factor activity, Metal ion binding, Nucleus, Regulation of DNA-templated transcription, Zinc ion binding |
| *nhr-21* | DNA-binding transcription factor activity, Regulation of DNA-templated transcription, Zinc ion binding, Metal ion binding, Nucleus |
| *sydn-1* | mRNA processing, Nuclear speck, Nucleus |
| *cyp-35B1* | Monooxygenase activity, Organic acid metabolic process, Oxidoreductase activity, Steroid hydroxylase activity, Xenobiotic metabolic process, Heme binding, Intracellular membrane-bounded organelle, Iron ion binding, Metal ion binding |
| *cyp-35A1* | Monooxygenase activity, Organic acid metabolic process, Oxidoreductase activity, Steroid hydroxylase activity, Xenobiotic metabolic process, Heme binding, Intracellular membrane-bounded organelle, Iron ion binding, Metal ion binding |
| *cyp-33C6* | Monooxygenase activity, Organic acid metabolic process, Oxidoreductase activity, Steroid hydroxylase activity, Xenobiotic metabolic process, Heme binding, Intracellular membrane-bounded organelle, Iron ion binding, Metal ion binding |
| *cyp-35B2* | Monooxygenase activity, Organic acid metabolic process, Oxidoreductase activity, Steroid hydroxylase activity, Xenobiotic metabolic process, Heme binding, Intracellular membrane-bounded organelle, Iron ion binding, Metal ion binding |
| *cyp-35B3* | Monooxygenase activity, Organic acid metabolic process, Oxidoreductase activity, Steroid hydroxylase activity, Xenobiotic metabolic process, Heme binding, Intracellular membrane-bounded organelle, Iron ion binding, Metal ion binding |
| *cyp-35A5* | Monooxygenase activity, Organic acid metabolic process, Oxidoreductase activity, Steroid hydroxylase activity, Xenobiotic metabolic process, Heme binding, Intracellular membrane-bounded organelle, Iron ion binding, Metal ion binding |
| *cyp-34A10* | Monooxygenase activity, Organic acid metabolic process, Oxidoreductase activity, Steroid hydroxylase activity, Xenobiotic metabolic process, Heme binding, Intracellular membrane-bounded organelle, Iron ion binding, Metal ion binding |
| *cyp-35A3* | Monooxygenase activity, Organic acid metabolic process, Oxidoreductase activity, Steroid hydroxylase activity, Xenobiotic metabolic process, Heme binding, Intracellular membrane-bounded organelle, Iron ion binding, Metal ion binding |
| *cyp-35C1* | Monooxygenase activity, Organic acid metabolic process, Oxidoreductase activity, Steroid hydroxylase activity, Xenobiotic metabolic process, Heme binding, Intracellular membrane-bounded organelle, Iron ion binding, Metal ion binding |
| *cyp-35A4* | Monooxygenase activity, Organic acid metabolic process, Oxidoreductase activity, Steroid hydroxylase activity, Xenobiotic metabolic process, Heme binding, Intracellular membrane-bounded organelle, Iron ion binding, Metal ion binding, |
| *cyp-13A6* | Monooxygenase activity, Oxidoreductase activity, Heme binding, Iron ion binding, Metal ion binding |
| *C08D8.1* | Membrane |
| *F12A10.1* | Membrane |
| *WBGene00018737* | N.A. |
| *Y105C5B.5* | N.A. |
| *R09E12.9* | N.A. |
| *AC3.9* | N.A. |
| *R02C2.7* | N.A. |
| *T19B10.2* | N.A. |
| *F59B1.2* | N.A. |
| *clec-223* | N.A. |
| *W03G1.5* | N.A. |
| *T12D8.5* | N.A. |

**Table S4**

| **Gene** | **Gene Ontology Association** |
| --- | --- |
| *dhs-18* | Mitochondrion |
| *thn-2* | Defense response to Gram-negative bacterium, Defense response to Gram-positive bacterium |
| *clec-7* | Defense response to Gram-positive bacterium |
| *acs-7* | ATP binding, catalytic activity, CoA-ligase activity, Fatty acid biosynthetic process, Medium-chain fatty acid-CoA ligase activity, Peroxisome, Ascaroside biosynthetic process |
| *cyp-13A7* | Oxidoreductase activity, Heme binding, Iron ion binding, Metal ion binding, Monooxygenase activity |
| *Y51H4A.5* | Lipid metabolic process |
| *F11D5.5* | Hexose transmembrane transport, Membrane, Monosaccharide transmembrane transport, Transmembrane transport |
| *ZC190.4* | Negative regulation of translation, Translation repressor activity, Nuclear-transcribed mRNA poly(A) tail shortening, mRNA binding, P-body, RNA binding |
| *Y47H10A.5* | Metal ion binding, mRNA 5'-diphosphatase activity, NAD-cap decapping, Nuclear-transcribed mRNA catabolic process, Nuclease activity, Nucleotide binding, Nucleus, RNA binding |
| *ets-9* | DNA binding, DNA-binding transcription factor activity, Regulation of DNA-templated transcription, Regulation of transcription by RNA polymerase II, Nucleus |
| *Y51B9A.9* | ATP binding, Intracellular signal transduction, Kinase activity, MAP kinase activity, MAPK cascade, Nucleus, Protein phosphorylation |
| *Y53F4B.1* | Response to stress, ATP binding, Necroptotic signaling pathway, Phosphorylation, Protein serine/threonine kinase activity |
| *trpl-2* | Membrane, Metal ion transport, Monoatomic cation channel activity, Monoatomic cation transmembrane transport |
| *H04D03.4* | Cul2-RING ubiquitin ligase complex |
| *T05E12.3* | Protein homooligomerization |
| *tag-234* | N.A. |
| *F10C2.3* | N.A. |
| *fbxa-54* | N.A. |

**Table S5**

| **Gene** | **Gene Ontology Association** |
| --- | --- |
| *ZK550.6* | Fatty acid alpha-oxidation, Fatty acid metabolic process, L-ascorbic acid binding, Metal ion binding, Phytanoyl-CoA dioxygenase activity, Dioxygenase activity |
| *F41E6.5* | Fatty acid alpha-oxidation, FMN binding, Oxidoreductase activity, Peroxisomal matrix, Peroxisome, (S)-2-hydroxy-acid oxidase activity |
| *Y48A6B.9* | Fatty acid biosynthetic process, Fatty acid metabolic process, Mitochondrion, Oxidoreductase activity, Enoyl-[acyl-carrier-protein] reductase (NADPH) activity |

**Table S6**

| Batch | Strain | Treatment | Median lifespan (days) | Mean lifespan ± SE (days) | | | Variation compared to control (%) | *P*-values against control | *P*-values summary | NTOTAL |
| --- | --- | --- | --- | --- | --- | --- | --- | --- | --- | --- |
| 1 | wild type (N2) | DMSO | 21 | 20.71 | ± | 0.43 | 0 |  |  | 105 |
|  |  | Terbinafine 31.25 µM | 23 | 22.06 | ± | 0.42 | 6.5 | <0.01 | ** | 131 |
|  |  | Miglustat 250 µM | 23 | 22.42 | ± | 0.49 | 8.3 | <0.01 | ** | 99 |
|  | *atfs-1* (gk3094) | DMSO | 19 | 19.04 | ± | 0.42 | 0 |  |  | 129 |
|  |  | Terbinafine 31.25 µM | 17 | 18.06 | ± | 0.47 | -5.2 | 0.1309 | ns | 103 |
|  |  | Miglustat 250 µM | 17 | 17.84 | ± | 0.52 | -6.3 | 0.0418 | * | 76 |
|  | *daf-16* (mu86) | DMSO | 15 | 16.55 | ± | 0.29 | 0 |  |  | 125 |
|  |  | Terbinafine 31.25 µM | 15 | 16.14 | ± | 0.34 | -2.5 | 0.6454 | ns | 100 |
|  |  | Miglustat 250 µM | 15 | 15.90 | ± | 0.33 | -4.0 | 0.2547 | ns | 108 |
|  | *daf-2* (e1370) | DMSO | 44 | 42.32 | ± | 1.15 | 0 |  |  | 155 |
|  |  | Terbinafine 31.25 µM | 44 | 42.15 | ± | 1.30 | 0 | 0.3133 | ns | 99 |
|  |  | Miglustat 250 µM | 44 | 42.26 | ± | 1.22 | -0.2 | 0.4679 | ns | 117 |
| 2 | wild type (N2) | DMSO | 21 | 20.71 | ± | 0.43 | 0 |  |  | 77 |
|  |  | Terbinafine 31.25 µM | 23 | 22.06 | ± | 0.42 | 6.5 | 0.0032 | ** | 66 |
|  |  | Miglustat 250 µM | 23 | 22.42 | ± | 0.49 | 8.3 | <0.0001 | **** | 111 |
|  | *atfs-1* (gk3094) | DMSO | 19 | 18.36 | ± | 0.40 | 0 |  |  | 131 |
|  |  | Terbinafine 31.25 µM | 18 | 18.22 | ± | 0.42 | -0.7 | 0.8077 | ns | 112 |
|  |  | Miglustat 250 µM | 17 | 17.10 | ± | 0.50 | -6.9 | 0.1285 | ns | 101 |
|  | *daf-16* (mu86) | DMSO | 13 | 14.02 | ± | 0.53 | 0 | 0.1285 |  | 51 |
|  |  | Terbinafine 31.25 µM | 13 | 13.39 | ± | 0.51 | -4.5 | 0.2998 | ns | 57 |
|  |  | Miglustat 250 µM | 13 | 13.21 | ± | 0.38 | -5.8 | 0.0605 | ns | 78 |
| 3 | wild type (N2) | DMSO | 21 | 21.14 | ± | 0.55 | 0 |  |  | 102 |
|  |  | Terbinafine 31.25 µM | 23 | 22.74 | ± | 0.74 | 7.6 | 0.0423 | * | 69 |
|  |  | Miglustat 250 µM | 25 | 23.82 | ± | 0.57 | 12.7 | 0.0003 | *** | 106 |
|  | *daf-16* (mu86) | DMSO | 13 | 12.73 | ± | 0.49 | 0 |  |  | 125 |
|  |  | Terbinafine 31.25 µM | 11 | 11.84 | ± | 0.54 | -7.0 | 0.1486 | ns | 100 |
|  |  | Miglustat 250 µM | 11 | 11.85 | ± | 0.47 | -6.9 | 0.1107 | ns | 108 |
|  | *daf-2* (e1370) | DMSO | 44 | 44.43 | ± | 1.13 | 0 |  |  | 134 |
|  |  | Terbinafine 31.25 µM | 44 | 43.14 | ± | 1.29 | -3 | 0.4571 | ns | 118 |
|  |  | Miglustat 250 µM | 44 | 42.80 | ± | 1.09 | -3.7 | 0.0582 | ns | 147 |

**Table S7**

| **Primer** | **Sequence** |
| --- | --- |
| ATF4-Fw: | TGAAGGAGTTCGACTTGGATGCC |
| ATF4-Rv: | CAGAAGGTCATCTGGCATGGTTTC |
| ATF5_Fw: | GAGCCCCTGGCAGGTGAT |
| ATF5_Rv: | CAGAGGGAGGAGAGCTGTGAA |
| CHOP_Fw: | ACAGTGTCCCGAAGGAGAAAGG |
| CHOP_Rv: | GCCAAAATCAGAGCTGGAACCT |
| MFN1-Fw: | GTGGCACTTGCTGAAGGATT |
| MFN1-Rv: | GTTTTCACTGCTGACTGCGA |
| MFN2_Fw: | GCGCTCGCTGGTGACGTAGT |
| MFN2_Rv: | GGGTAGGCATCATGGACATGGCT |
| TFAM_Fw: | CCAAAAAGACCTCGTTCAGC |
| TFAM_Rv: | TCCAGTTTTCCTTTACAGTCTTCAG |
| PARK2_Fw: | GTCAGGTTCAACTCCAGCCA |
| PARK2_Rv: | CACAATTCTGCACAGTCCAGTC |
| PINK1_Fw: | GATGAGGCTGGACTGAGGAG |
| PINK1_Rv: | TCCCACTCCCGTAACTGAAC |
| BNIP3_Fw: | AGGGCTCCTGGGTAGAACTG |
| BNIP3_Rv: | CCCTGTTGGTATCTTGTGGTG |
| GAPDH_Fw: | TTGGTATCGTGGAAGGACTC |
| GAPDH_Rv: | ACAGTCTTCTGGGTGGCAGT |

**Table S8**

| **Protein** | **Antibody reference** |
| --- | --- |
| LONP | HPA002192-100UL, Sigma |
| ASNS | HPA029318, Atlas antibodies |
| ATF4 | PA5-105288, Invitrogen |
| ATF5 | ab184923, Abcam |
| CHOP | ab11419, Abcam |
| ND1 | ab74257, Abcam |
| ND3 | MBS9202094, Mybiosource |
| ND6 | PA5-75196, Invitrogen |
| Total OXPHOS rodent WB antibody cocktail | #ab110413, Abcam |

**Table S9**
