# Supplementary material for: Targeting Mitochondrial Stress Responses: Terbinafine and Miglustat as Novel Lifespan and Healthspan Modulators
Source: Aging Cell. 2026 Mar 30;25(4):e70452. doi: 10.1111/acel.70452 (PMC13092511; doi:10.1111/acel.70452)
Supplement: Supplementary file 1 — Figure S1: Dose–response analysis of the top 18 compounds in hsp‐6p::gfp worms. hsp‐6p::gfp reporter strain was raised on NGM supplemented by vehicle (1% DMSO) or various concentrations of the compounds (31.25, 62.5, 125, 250, 500, and 1000 μM). Empty panels represent toxic conditions. Drug names in blue indicate selected compounds and arrows indicate the concentrations selected for further analysis. Scale bars, 0.3 mm. Figure S2: Lifespan measurements of worms treated with the selected compounds. x‐axis shows lifespan in days. y‐axis shows the percentage of worms alive. Statistics are reported in Table S2. Figure S3: Evaluating the impact of terbinafine and miglustat on development and reproductive capacity. (A) Egg‐laying rate of adult worms following doxycycline (50 μM), terbinafine (31.25 μM), miglustat (250 μM) treatment. Reproductive capacity is measured by the number of eggs laid by animal per hour (No. of eggs/animal/h). Significance was determined using two‐way ANOVA with Tukey correction (*p < 0.05, **p < 0.01, ***p < 0.001). (B, C) Worm body size at Day 1 adult stage following various concentrations of terbinafine (B) and miglustat (C). Body size is characterized by the perimeter (mm) and the area (mm2), and the animal morphology by the area‐to‐perimeter ratio. Significance was determined using one way ANOVA with Bonferroni correction (*p < 0.05, **p < 0.01, ***p < 0.001). Figure S4: Evaluating the impact of terbinafine and miglustat on mitophagy. (A) Heatmap of the expression of mitophagy related‐genes following treatment with doxycycline (50 μM), terbinafine (31.25 μM), miglustat (250 μM) versus control. Expression values are represented in log2 fold changes (log2FC). Statistical significance after FDR correction for multiple testing are indicated as follow: *p < 0.1, **p < 0.05, ***p < 0.01. (B) Quantitative RT‐PCR analysis of mitochondrial biogenesis, mitophagy and autophagy related‐genes, in C. elegans following treatment with terbinafine (15 μM, 31. [file ACEL-25-e70452-s001.pdf]

Figure S1

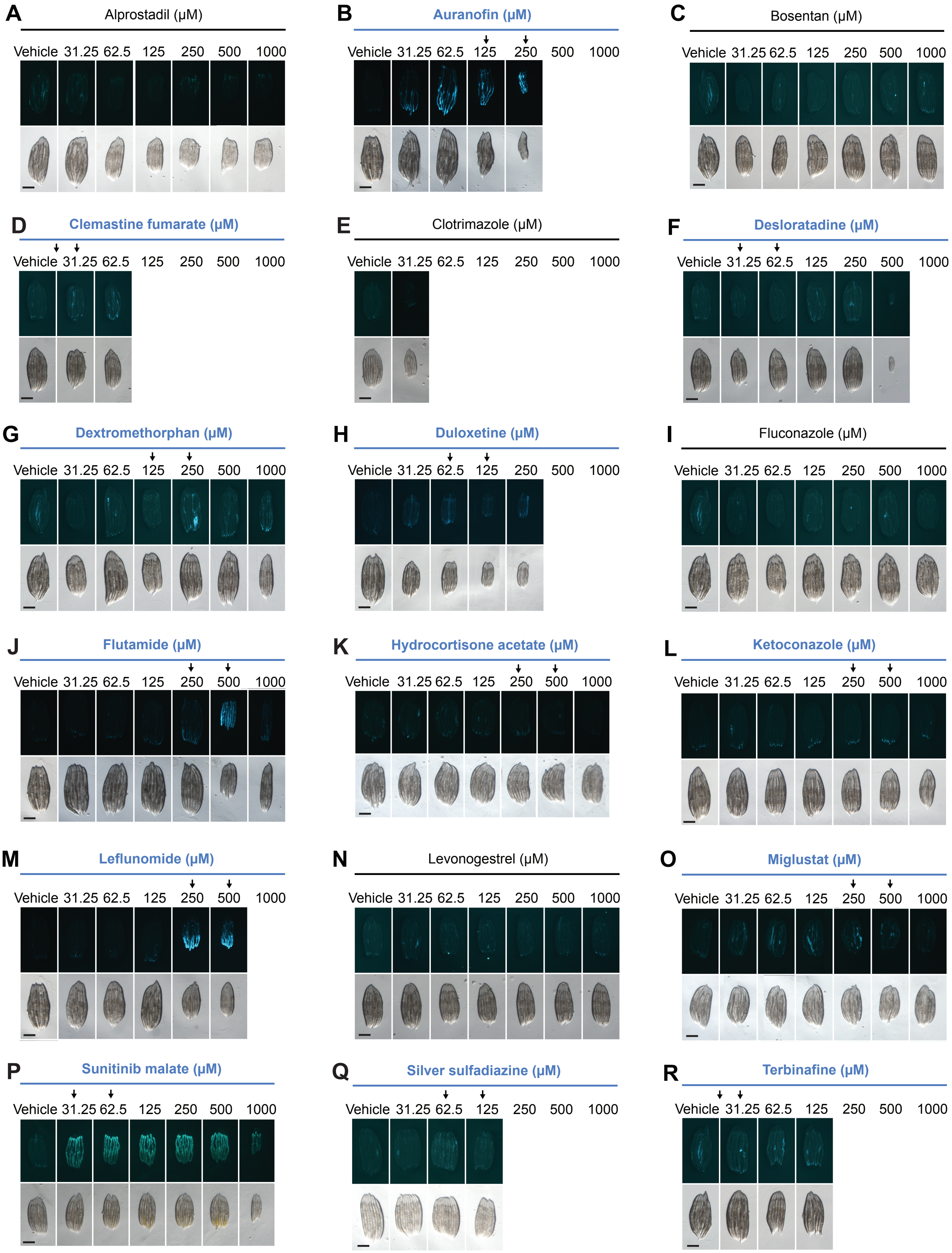
A
Alprostadil (μM)
Vehicle 31.25 62.5 125 250 500 1000
B
Auranofin (μM)
Vehicle 31.25 62.5 125 250 500 1000
C
Bosentan (μM)
Vehicle 31.25 62.5 125 250 500 1000
D
Clemastine fumarate (μM)
Vehicle 31.25 62.5 125 250 500 1000
E
Clotrimazole (μM)
Vehicle 31.25 62.5 125 250 500 1000
F
Desloratadine (μM)
Vehicle 31.25 62.5 125 250 500 1000
G
Dextromethorphan (μM)
Vehicle 31.25 62.5 125 250 500 1000
H
Duloxetine (μM)
Vehicle 31.25 62.5 125 250 500 1000
I
Fluconazole (μM)
Vehicle 31.25 62.5 125 250 500 1000
J
Flutamide (μM)
Vehicle 31.25 62.5 125 250 500 1000
K
Hydrocortisone acetate (μM)
Vehicle 31.25 62.5 125 250 500 1000
L
Ketoconazole (μM)
Vehicle 31.25 62.5 125 250 500 1000
M
Leflunomide (μM)
Vehicle 31.25 62.5 125 250 500 1000
N
Levonogestrel (μM)
Vehicle 31.25 62.5 125 250 500 1000
O
Miglustat (μM)
Vehicle 31.25 62.5 125 250 500 1000
P
Sunitinib malate (μM)
Vehicle 31.25 62.5 125 250 500 1000
Q
Silver sulfadiazine (μM)
Vehicle 31.25 62.5 125 250 500 1000
R
Terbinafine (μM)
Vehicle 31.25 62.5 125 250 500 1000

Figure S2

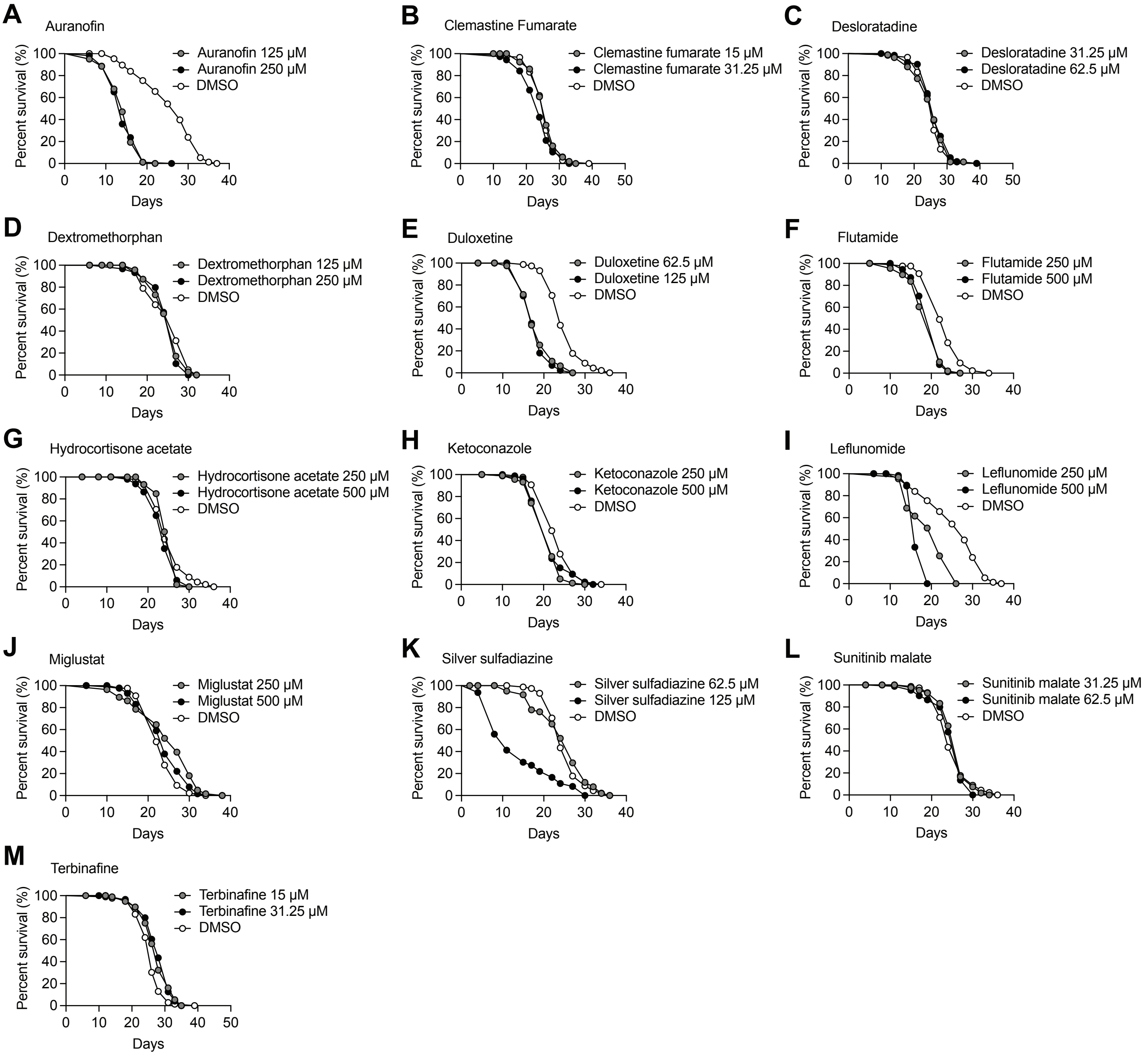
A
Auranofin
Auranofin 125 µM
Auranofin 250 µM
DMSO
B
Clemastine Fumarate
Clemastine fumarate 15 µM
Clemastine fumarate 31.25 µM
DMSO
C
Desloratadine
Desloratadine 31.25 µM
Desloratadine 62.5 µM
DMSO
D
Dextromethorphan
Dextromethorphan 125 µM
Dextromethorphan 250 µM
DMSO
E
Duloxetine
Duloxetine 62.5 µM
Duloxetine 125 µM
DMSO
F
Flutamide
Flutamide 250 µM
Flutamide 500 µM
DMSO
G
Hydrocortisone acetate
Hydrocortisone acetate 250 µM
Hydrocortisone acetate 500 µM
DMSO
H
Ketoconazole
Ketoconazole 250 µM
Ketoconazole 500 µM
DMSO
I
Leflunomide
Leflunomide 250 µM
Leflunomide 500 µM
DMSO
J
Miglustat
Miglustat 250 µM
Miglustat 500 µM
DMSO
K
Silver sulfadiazine
Silver sulfadiazine 62.5 µM
Silver sulfadiazine 125 µM
DMSO
L
Sunitinib malate
Sunitinib malate 31.25 µM
Sunitinib malate 62.5 µM
DMSO
M
Terbinafine
Terbinafine 15 µM
Terbinafine 31.25 µM
DMSO
Percent survival (%)
Days

Figure S3

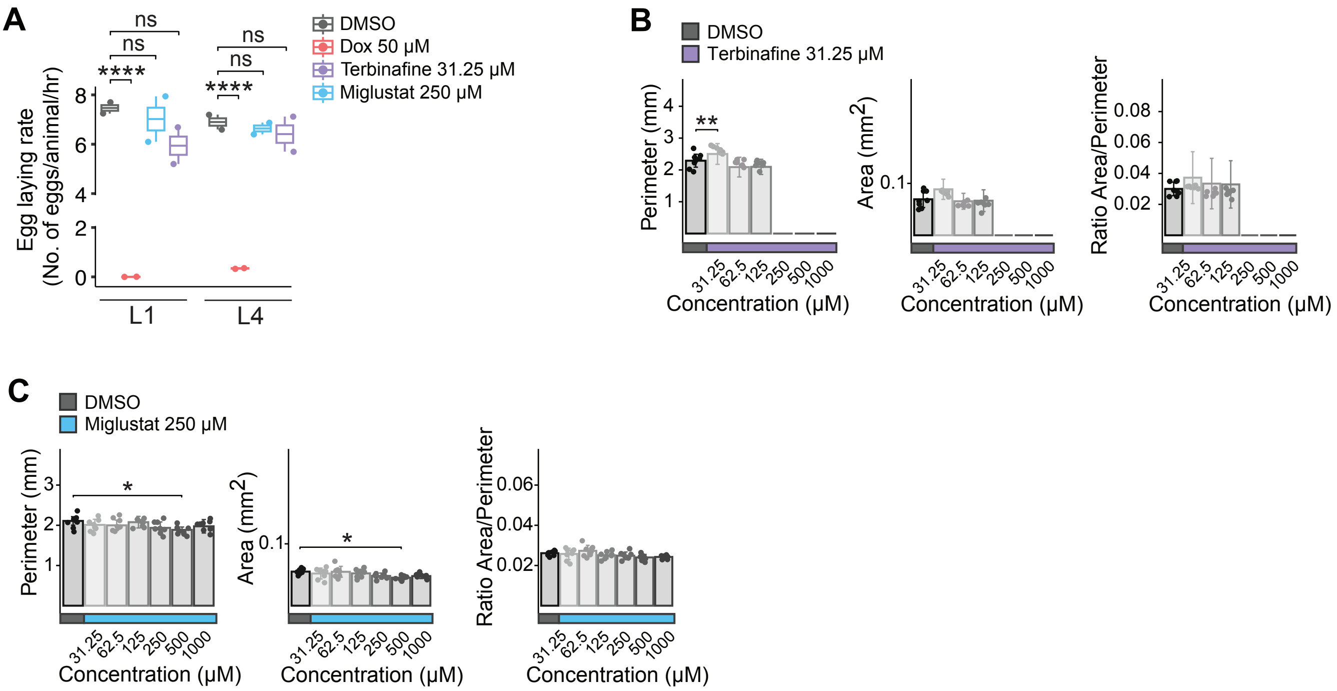

A
DMSO
Dox 50 μM
Terbinafine 31.25 μM
Miglustat 250 μM
Egg laying rate
(No. of eggs/animal/hr)
ns
ns
****
L1
L4
B
DMSO
Terbinafine 31.25 μM
Perimeter (mm)
Area (mm2)
Ratio Area/Perimeter
**
31.25 62.5 125 250 500 1000
Concentration (μM)
C
DMSO
Miglustat 250 μM
*

Figure S4

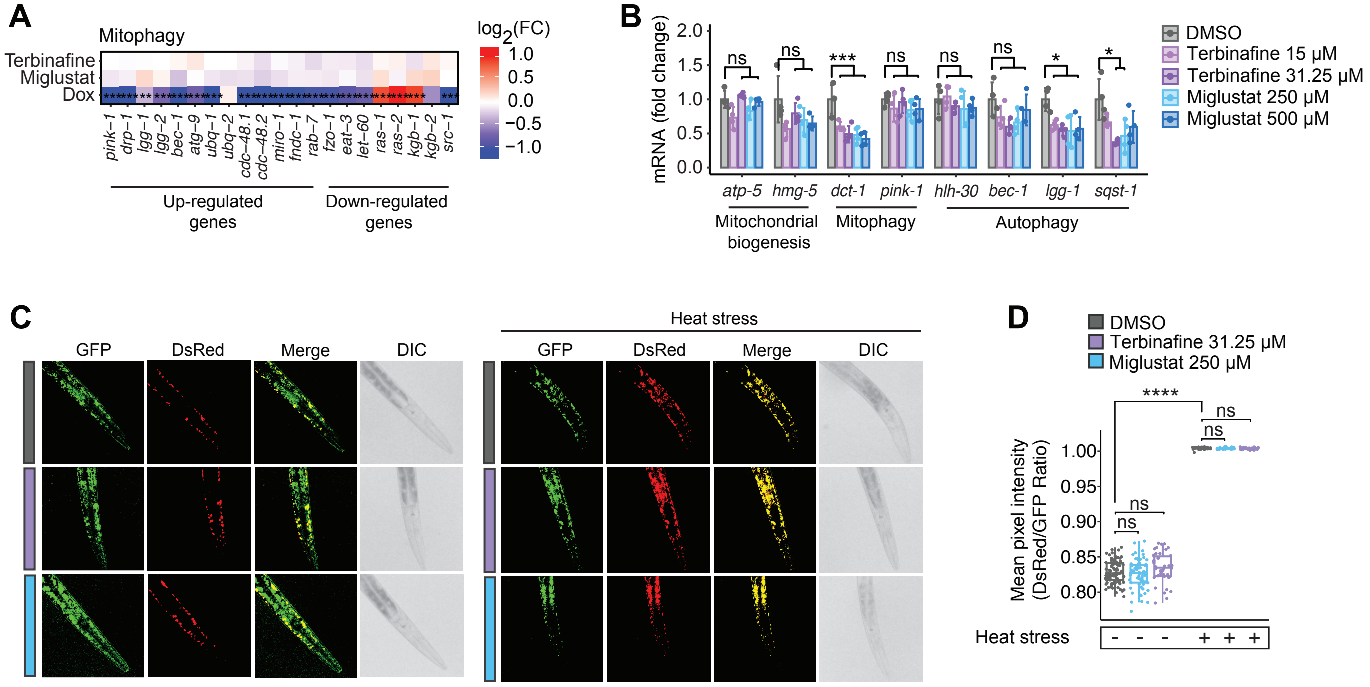

A
Mitophagy
Terbinafine
Miglustat
Dox
log2(FC)
1.0
0.5
0.0
−0.5
−1.0
pink−1 drp−1 lgg−1 lgg−2 bec−1 atg−9 ubq−1 ubq−2 cdc−48.1 cdc−48.2 miro−1 fndc−1 rab−7 fzo−1 eat−3 let−60 ras−1 ras−2 kgb−1 kgb−2 src−1
Up-regulated genes
Down-regulated genes
B
mRNA (fold change)
DMSO
Terbinafine 15 μM
Terbinafine 31.25 μM
Miglustat 250 μM
Miglustat 500 μM
atp-5 hmg-5 dct-1 pink-1 hlh-30 bec-1 lgg-1 sqst-1
Mitochondrial biogenesis
Mitophagy
Autophagy
C
Heat stress
GFP DsRed Merge DIC
D
DMSO
Terbinafine 31.25 μM
Miglustat 250 μM
Mean pixel intensity (DsRed/GFP Ratio)
Heat stress

Figure S5

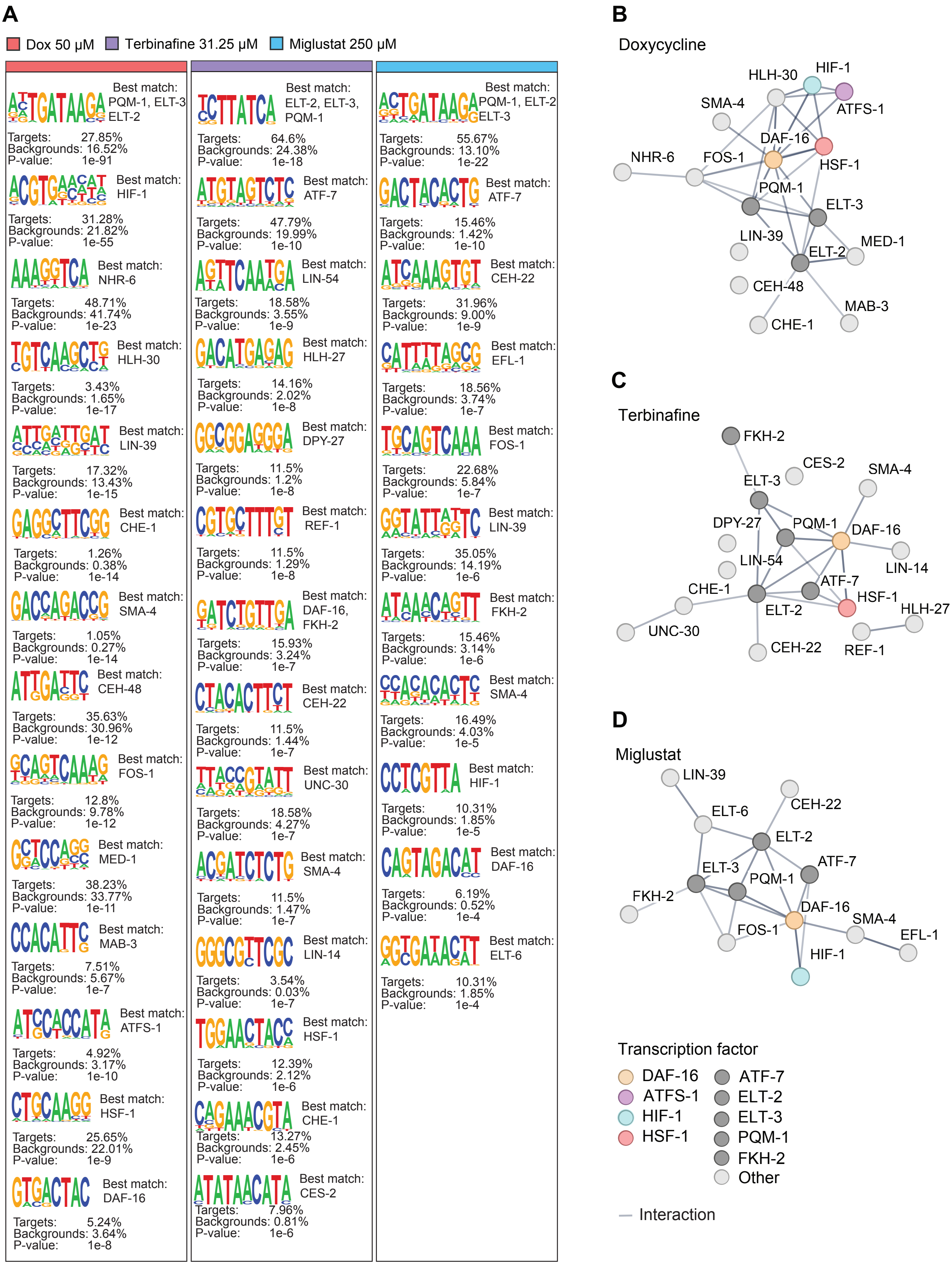

A
Dox 50 μM
Terbinafine 31.25 μM
Miglustat 250 μM
Best match: PQM-1, ELT-3 ELT-2
Targets: 27.85%
Backgrounds: 16.52%
P-value: 1e-91
Best match: HIF-1
Targets: 31.28%
Backgrounds: 21.82%
P-value: 1e-55
Best match: NHR-6
Targets: 48.71%
Backgrounds: 41.74%
P-value: 1e-23
Best match: HLH-30
Targets: 3.43%
Backgrounds: 1.65%
P-value: 1e-17
Best match: LIN-39
Targets: 17.32%
Backgrounds: 13.43%
P-value: 1e-15
Best match: CHE-1
Targets: 1.26%
Backgrounds: 0.38%
P-value: 1e-14
Best match: SMA-4
Targets: 1.05%
Backgrounds: 0.27%
P-value: 1e-14
Best match: CEH-48
Targets: 35.63%
Backgrounds: 30.96%
P-value: 1e-12
Best match: FOS-1
Targets: 12.8%
Backgrounds: 9.78%
P-value: 1e-12
Best match: MED-1
Targets: 38.23%
Backgrounds: 33.77%
P-value: 1e-11
Best match: MAB-3
Targets: 7.51%
Backgrounds: 5.67%
P-value: 1e-7
Best match: ATFS-1
Targets: 4.92%
Backgrounds: 3.17%
P-value: 1e-10
Best match: HSF-1
Targets: 25.65%
Backgrounds: 22.01%
P-value: 1e-9
Best match: DAF-16
Targets: 5.24%
Backgrounds: 3.64%
P-value: 1e-8
Best match: ELT-2, ELT-3, PQM-1
Targets: 64.6%
Backgrounds: 24.38%
P-value: 1e-18
Best match: ATF-7
Targets: 47.79%
Backgrounds: 19.99%
P-value: 1e-10
Best match: LIN-54
Targets: 18.58%
Backgrounds: 3.55%
P-value: 1e-9
Best match: HLH-27
Targets: 14.16%
Backgrounds: 2.02%
P-value: 1e-8
Best match: DPY-27
Targets: 11.5%
Backgrounds: 1.2%
P-value: 1e-8
Best match: REF-1
Targets: 11.5%
Backgrounds: 1.29%
P-value: 1e-8
Best match: DAF-16, FKH-2
Targets: 15.93%
Backgrounds: 3.24%
P-value: 1e-7
Best match: CEH-22
Targets: 11.5%
Backgrounds: 1.44%
P-value: 1e-7
Best match: UNC-30
Targets: 18.58%
Backgrounds: 4.27%
P-value: 1e-7
Best match: SMA-4
Targets: 11.5%
Backgrounds: 1.47%
P-value: 1e-7
Best match: LIN-14
Targets: 3.54%
Backgrounds: 0.03%
P-value: 1e-7
Best match: HSF-1
Targets: 12.39%
Backgrounds: 2.12%
P-value: 1e-6
Best match: CHE-1
Targets: 13.27%
Backgrounds: 2.45%
P-value: 1e-6
Best match: CES-2
Targets: 7.96%
Backgrounds: 0.81%
P-value: 1e-6
Best match: PQM-1, ELT-2 ELT-3
Targets: 55.67%
Backgrounds: 13.10%
P-value: 1e-22
Best match: ATF-7
Targets: 15.46%
Backgrounds: 1.42%
P-value: 1e-10
Best match: CEH-22
Targets: 31.96%
Backgrounds: 9.00%
P-value: 1e-9
Best match: EFL-1
Targets: 18.56%
Backgrounds: 3.74%
P-value: 1e-7
Best match: FOS-1
Targets: 22.68%
Backgrounds: 5.84%
P-value: 1e-7
Best match: LIN-39
Targets: 35.05%
Backgrounds: 14.19%
P-value: 1e-6
Best match: FKH-2
Targets: 15.46%
Backgrounds: 3.14%
P-value: 1e-6
Best match: SMA-4
Targets: 16.49%
Backgrounds: 4.03%
P-value: 1e-5
Best match: HIF-1
Targets: 10.31%
Backgrounds: 1.85%
P-value: 1e-5
Best match: DAF-16
Targets: 6.19%
Backgrounds: 0.52%
P-value: 1e-4
Best match: ELT-6
Targets: 10.31%
Backgrounds: 1.85%
P-value: 1e-4
B
Doxycycline
HLH-30
HIF-1
SMA-4
ATFS-1
DAF-16
NHR-6
FOS-1
HSF-1
PQM-1
ELT-3
LIN-39
MED-1
ELT-2
CEH-48
MAB-3
CHE-1
C
Terbinafine
FKH-2
CES-2
SMA-4
ELT-3
DPY-27
PQM-1
DAF-16
LIN-14
LIN-54
ATF-7
CHE-1
HSF-1
HLH-27
ELT-2
UNC-30
CEH-22
REF-1
D
Miglustat
LIN-39
CEH-22
ELT-6
ELT-2
ATF-7
ELT-3
PQM-1
FKH-2
DAF-16
SMA-4
FOS-1
EFL-1
HIF-1
Transcription factor
DAF-16
ATFS-1
HIF-1
HSF-1
ATF-7
ELT-2
ELT-3
PQM-1
FKH-2
Other
Interaction

Figure S6

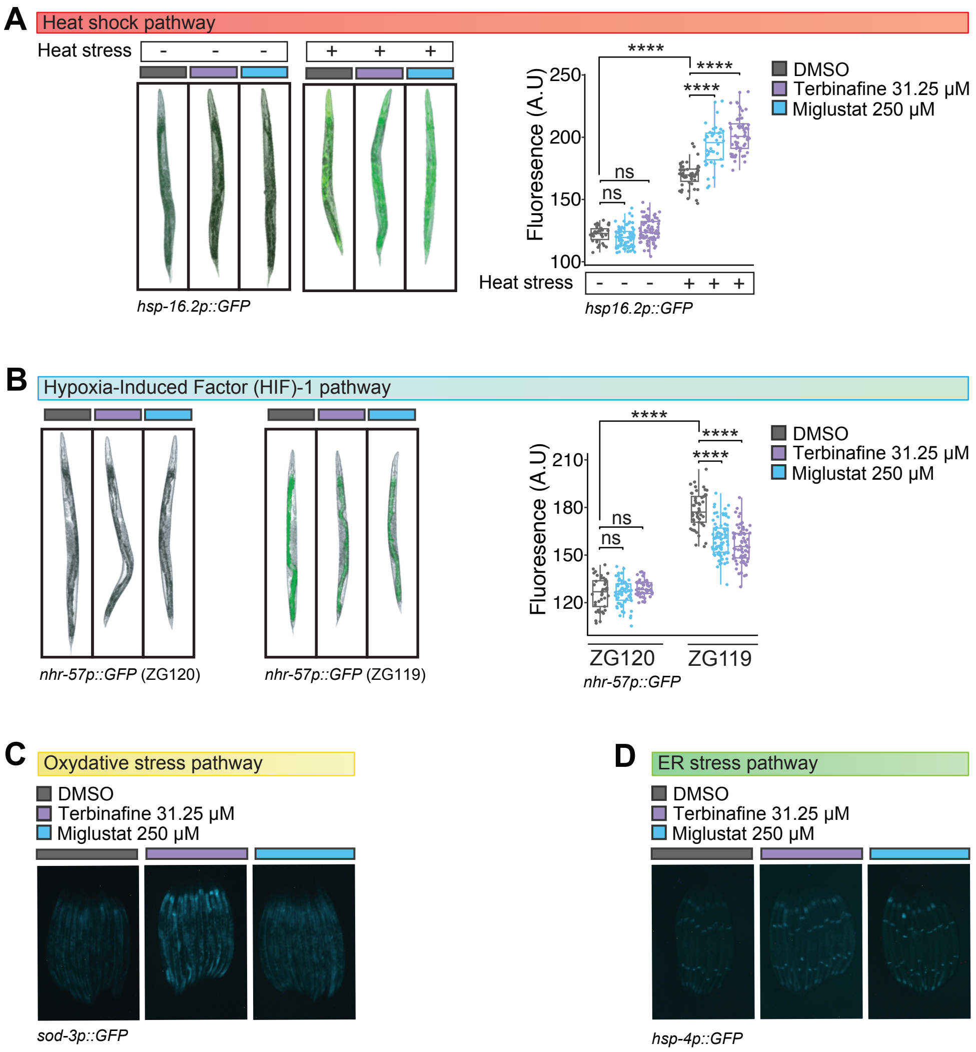

A
Heat shock pathway
Heat stress
- - -
+ + +
hsp-16.2p::GFP
****
****
****
ns
ns
Fluoresence (A.U)
250
200
150
100
Heat stress
- - - + + +
hsp16.2p::GFP
DMSO
Terbinafine 31.25 µM
Miglustat 250 µM
B
Hypoxia-Induced Factor (HIF)-1 pathway
nhr-57p::GFP (ZG120)
nhr-57p::GFP (ZG119)
****
****
****
ns
ns
Fluoresence (A.U)
210
180
150
120
ZG120
ZG119
nhr-57p::GFP
DMSO
Terbinafine 31.25 µM
Miglustat 250 µM
C
Oxydative stress pathway
DMSO
Terbinafine 31.25 µM
Miglustat 250 µM
sod-3p::GFP
D
ER stress pathway
DMSO
Terbinafine 31.25 µM
Miglustat 250 µM
hsp-4p::GFP

Figure S7

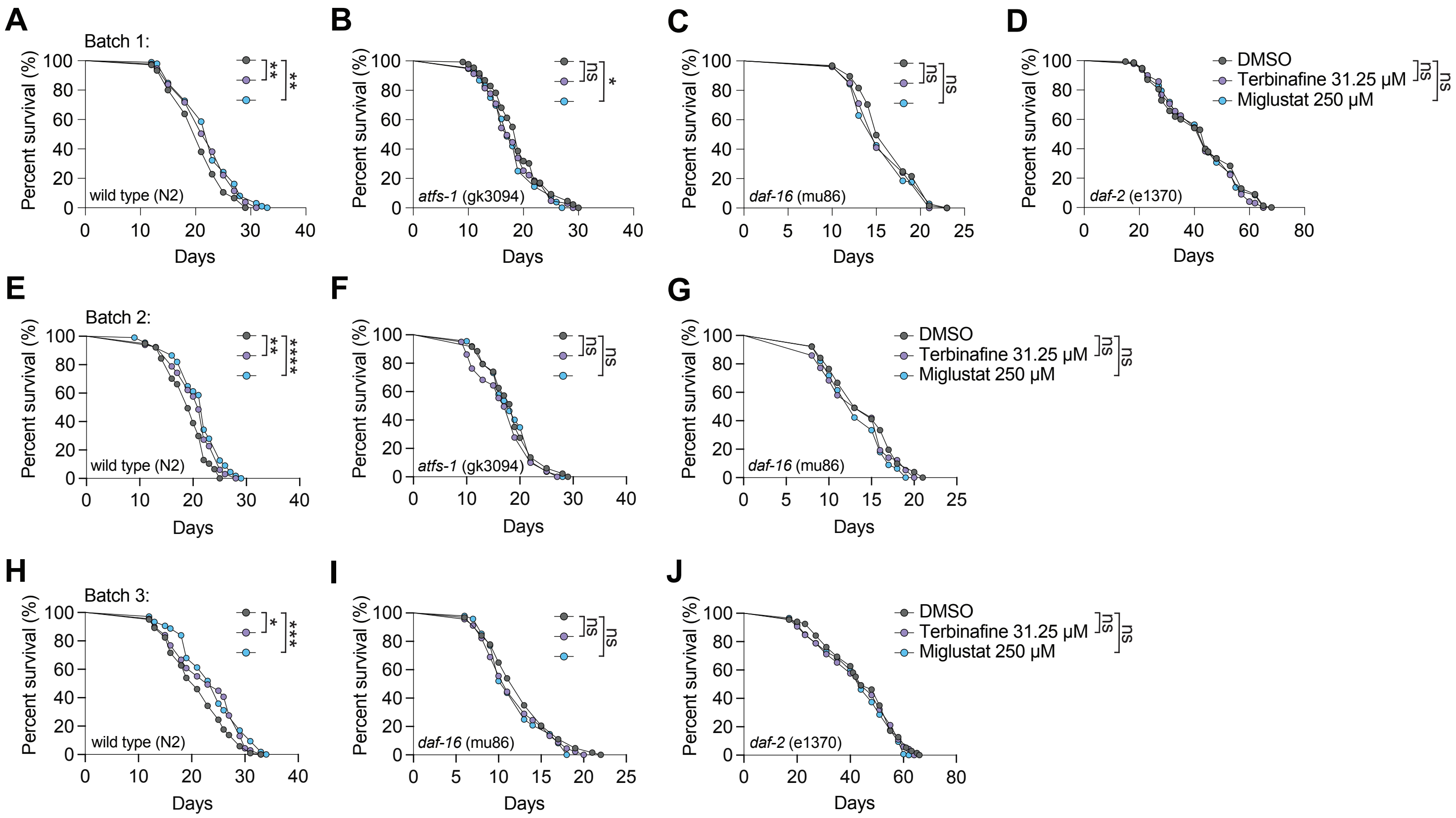

A
Batch 1:
Percent survival (%)
wild type (N2)
Days
B
atfs-1 (gk3094)
C
daf-16 (mu86)
D
daf-2 (e1370)
DMSO
Terbinafine 31.25 µM
Miglustat 250 µM
E
Batch 2:
F
G
H
Batch 3:
I
J
ns

Figure S8

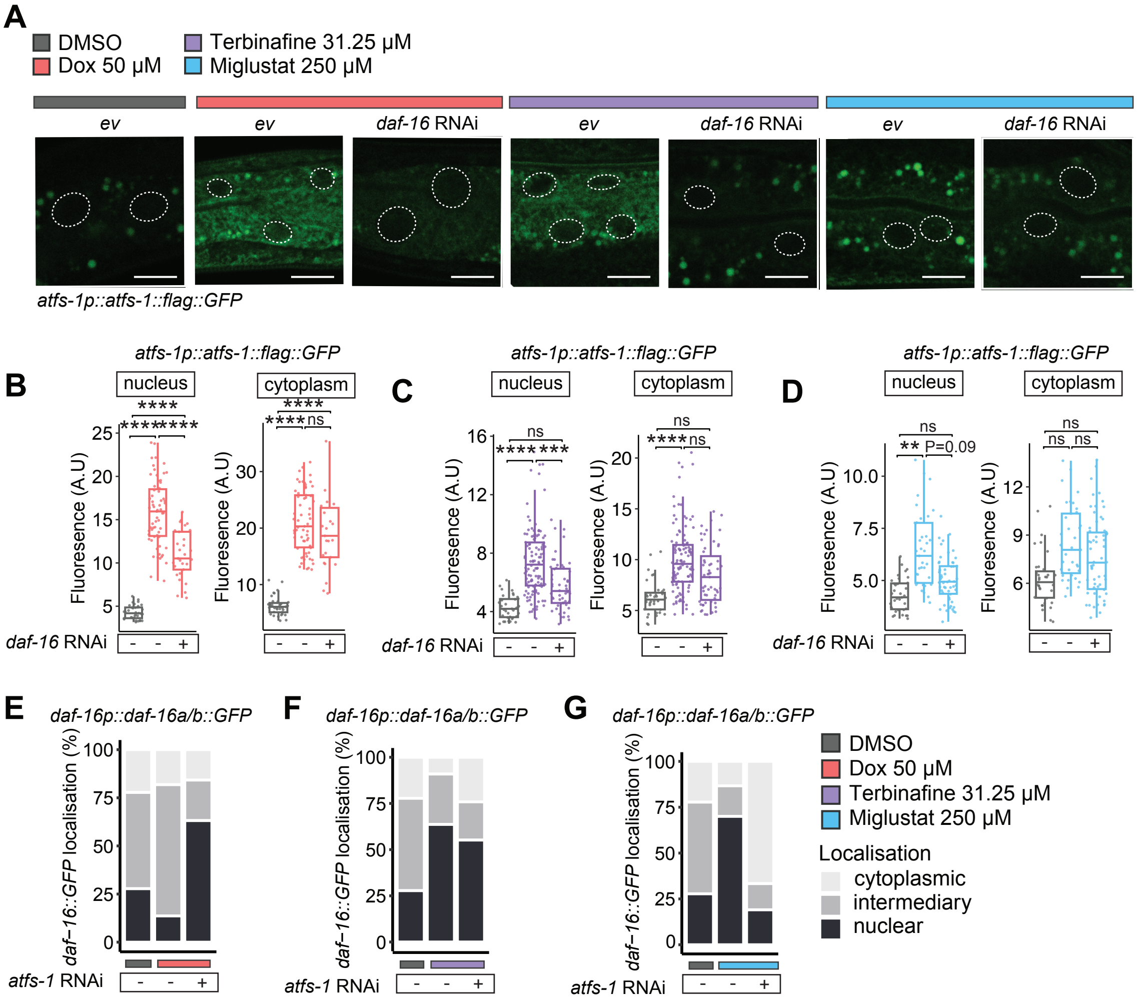
A
DMSO
Dox 50 μM
Terbinafine 31.25 μM
Miglustat 250 μM
ev
daf-16 RNAi
atfs-1p::atfs-1::flag::GFP
B
C
D
nucleus
cytoplasm
Fluoresence (A.U)
daf-16 RNAi
ns
P=0.09
E
F
G
daf-16p::daf-16a/b::GFP
daf−16::GFP localisation (%)
atfs-1 RNAi
Localisation
cytoplasmic
intermediary
nuclear

Figure S9

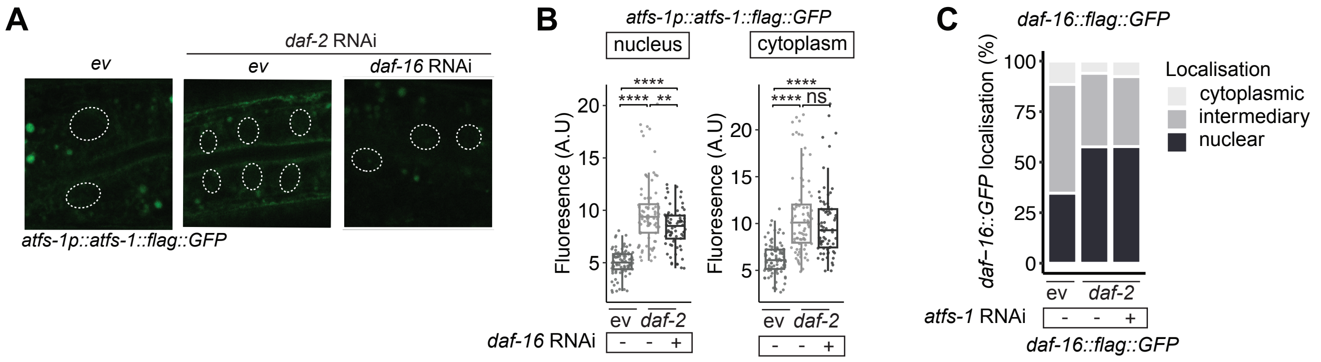

A
ev
daf-2 RNAi
ev
daf-16 RNAi
atfs-1p::atfs-1::flag::GFP
B
atfs-1p::atfs-1::flag::GFP
nucleus
cytoplasm
****
**** **
**** ns
Fluoresence (A.U)
ev daf-2
daf-16 RNAi
C
daf-16::flag::GFP
daf−16::GFP localisation (%)
Localisation
cytoplasmic
intermediary
nuclear
ev daf-2
atfs-1 RNAi
daf-16::flag::GFP

Figure S10

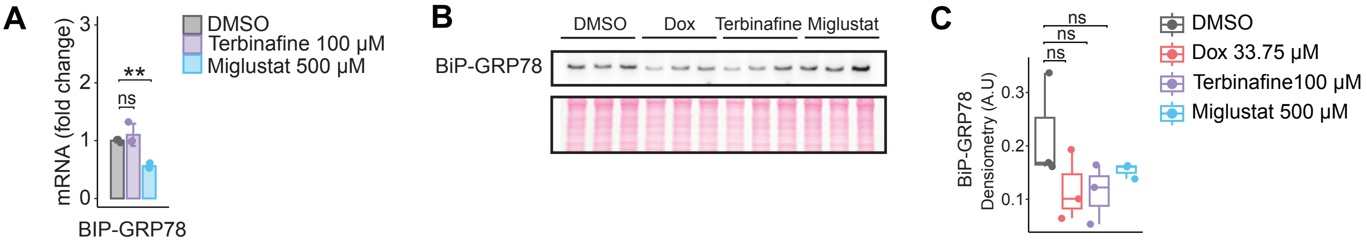
A
DMSO
Terbinafine 100 µM
Miglustat 500 µM
mRNA (fold change)
3
2
1
0
**
ns
BIP-GRP78
B
DMSO
Dox
Terbinafine
Miglustat
BiP-GRP78
C
ns
ns
ns
BiP-GRP78
Densiometry (A.U)
0.3
0.2
0.1
DMSO
Dox 33.75 µM
Terbinafine100 µM
Miglustat 500 µM
